# Efficacy and safety of 3-month versus 6-month oxaliplatin-based adjuvant chemotherapy in colorectal cancer: a systematic review and meta-analysis

**DOI:** 10.3389/fonc.2026.1762273

**Published:** 2026-02-02

**Authors:** Haiqiong Wu, Jun Li, Jidong Miao, Jiawei Li

**Affiliations:** 1 Department of Oncology, Zigong Fourth People’s Hospital, Zigong, Sichuan, China; 2 Department of Anesthesiology, Zigong Fourth People’s Hospital, Zigong, Sichuan, China

**Keywords:** adjuvant chemotherapy duration, CAPOX, colorectal cancer, FOLFOX, meta-analysis, peripheral neuropathy

## Abstract

**Background:**

The optimal duration of oxaliplatin-based adjuvant chemotherapy for stage II–III colorectal cancer (CRC) remains uncertain. Although a 3-month regimen may reduce toxicity, particularly peripheral sensory neuropathy (PSN), its effect on long-term survival is unclear. This study systematically compared the efficacy and safety of 3 versus 6 months of oxaliplatin-based adjuvant chemotherapy in stage II–III CRC patients.

**Methods:**

A systematic search of PubMed, Embase, the Cochrane Library, and Web of Science was conducted to identify randomized controlled trials (RCTs) comparing 3 months versus 6 months FOLFOX or CAPOX adjuvant chemotherapy in stage II–III CRC patients. Outcomes included disease-free survival (DFS), 3-year DFS rate, overall survival (OS), chemotherapy completion rate, and PSN. The certainty of evidence was evaluated using the GRADE approach, and trial sequential analysis (TSA) was performed to assess the robustness of the results.

**Results:**

Six RCTs involving a total of 16,420 stage II–III CRC patients were included. In the overall patients, the 3-month regimen showed no significant difference in DFS compared with the 6-month regimen (HR = 1.05, 95% CI 0.98–1.13, P = 0.18). Within the FOLFOX subgroup, the risk of DFS events was increased in the 3-month regimen (HR = 1.21, 95% CI 1.11–1.33, P < 0.001), with a similar difference observed among stage III patients (HR = 1.23, 95% CI 1.06–1.43, P = 0.007). No significant differences were observed in OS or 3-year OS rate, and all subgroup analyses showed no statistically significant differences in 3-year DFS rates. The 3-month regimen demonstrated a significantly higher chemotherapy completion rate, and grade 3–4 PSN was significantly lower in the 3 months regimen.

**Conclusion:**

Stage II patients may preferentially receive a 3-month treatment regimen, whereas stage III patients receiving FOLFOX should still consider a 6-month regimen.

**Systematic Review Registration:**

https://inplasy.com/inplasy-2025-12-0022/, identifier INPLASY-2025-12-0022.

## Introduction

Colorectal cancer (CRC) is one of the most common malignancies worldwide and remains a leading cause of cancer-related morbidity and mortality (1). In high-risk patients, postoperative adjuvant chemotherapy plays a crucial role in improving disease-free survival (DFS) and overall survival (OS) (2, 3). Oxaliplatin, a widely used platinum-based agent, has become a standard component of adjuvant treatment for patients with stage II and stage III CRC (4, 5). When combined with fluoropyrimidines (5-FU), oxaliplatin is typically administered through the FOLFOX or CAPOX regimens (6, 7). However, the optimal duration of oxaliplatin-based adjuvant chemotherapy remains a matter of debate (8).

The balance between efficacy and toxicity for the traditional 6-month regimen versus the shorter 3-month regimen has been widely discussed (9). In recent years, several randomized controlled trials (RCTs) have directly compared 3 months and 6 months of adjuvant chemotherapy (10–16). These studies indicate that, for some low-risk patients, shortening treatment duration may not compromise efficacy while significantly reducing severe adverse events and improving treatment adherence and quality of life. Against this background, multiple meta-analyses have evaluated the relative effectiveness of 3-month and 6-month regimens in stage II–III CRC (17). It is noteworthy, however, that considerable heterogeneity persists among studies. Most trials use hazard ratios (HRs) for DFS as primary endpoints. Therefore, adopting a fixed time point, such as the 3-year DFS rate, provides a more intuitive and comparable measure of prognosis across studies with differing follow-up periods. Based on this rationale, the present study expands upon prior meta-analyses by incorporating and comparing 3-year DFS rates, with particular emphasis on the impact of treatment duration on long-term outcomes. Furthermore, we applied the Grading of Recommendations Assessment, Development and Evaluation (GRADE) framework to assess the certainty of evidence and used trial sequential analysis (TSA) to evaluate the robustness and sufficiency of the cumulative data. The aim of this study is to provide evidence-based guidance for individualized adjuvant chemotherapy strategies in stage II and III CRC, especially regarding the optimal treatment duration and regimen selection, thereby supporting more precise clinical decision-making.

## Methods

This systematic review and meta-analysis was performed following the updated Preferred Reporting Items for Systematic Reviews and Meta-Analyses (PRISMA 2020) recommendations (18). The study protocol was prospectively registered in INPLASY (Registration number: INPLASY2025120022).

### Inclusion and exclusion criteria

#### Inclusion criteria

Studies were eligible if they met the following criteria: (1) RCTs; (2) enrolled patients with stage II or stage III CRC who received postoperative oxaliplatin-based adjuvant chemotherapy, including FOLFOX (fluorouracil + leucovorin + oxaliplatin) or CAPOX (capecitabine + oxaliplatin); (3) compared two treatment durations—3 months versus 6 months; and (4) reported at least one of the following outcomes: DFS, 3-year DFS rate, OS, 3-year OS rate, chemotherapy completion rate, or peripheral sensory neuropathy (PSN) of any grade (grade 1, grade 2, or grade 3–4).

#### Exclusion criteria

Studies were excluded if they met any of the following conditions: non-RCTs; studies that did not clearly report the duration of postoperative adjuvant chemotherapy or did not include oxaliplatin-based regimens (FOLFOX or CAPOX); studies in which the population was not restricted to stage II or stage III colorectal cancer; studies that failed to provide key outcomes such as DFS, OS, chemotherapy completion rate, or PSN; duplicate publications, conference abstracts, reviews, meta-analyses, case reports; and studies with incomplete or unavailable data that could not be included in the quantitative analysis.

### Search strategy

A systematic search was conducted in four electronic databases—PubMed, Embase, the Cochrane Library, and Web of Science—from their inception to December 2, 2024. Both Medical Subject Headings (MeSH/Emtree terms) and free-text terms were used. Disease-related search terms included “Colorectal Neoplasms,” “Rectal Neoplasms,” “colorectal cancer,” “colon cancer,” “rectal cancer,” “colorectal carcinoma,” “colorectal neoplasm,” “colon carcinoma,” and “rectal carcinoma.” Treatment-related terms included “Oxaliplatin,” “FOLFOX,” “CAPOX,” “XELOX,” “capecitabine plus oxaliplatin,” “fluoropyrimidine,” “Fluorouracil,” “5-FU,” and “capecitabine.” Terms related to chemotherapy duration included “3 months,” “3-month,” “three months,” “6 months,” “6-month,” “six months,” “3 versus 6,” and “duration.” Study design terms included “Randomized Controlled Trial,” “randomized,” “randomized,” and “RCT.” To ensure the comprehensiveness of the search, reference lists of included studies and relevant reviews were also screened to identify additional eligible trials. All literature searches, screening, and full-text assessments were performed independently by two investigators, with disagreements resolved through consultation with a third investigator.

### Data extraction

Data from all included RCTs were independently extracted by two investigators using a standardized data extraction form. Any discrepancies were resolved through discussion with a third investigator to reach consensus. Extracted information included: (1) basic study characteristics, such as first author, country, sample size, and baseline patient characteristics (e.g., age, sex, tumor stage); and (2) outcome measures, including DFS, 3-year DFS rate, OS, 3-year OS rate, chemotherapy completion rate, and PSN of different grades.

### Risk of bias assessment

The methodological quality of the included RCTs was evaluated using the Cochrane Risk of Bias Tool (ROB 1.0) recommended by the Cochrane Collaboration. This tool assesses seven domains of potential bias: (1) random sequence generation; (2) allocation concealment; (3) blinding of participants and personnel; (4) blinding of outcome assessment; (5) completeness of outcome data; (6) selective reporting; and (7) other sources of bias (19). Two investigators independently performed the risk-of-bias assessment, and any disagreements were resolved through discussion or adjudication by a third investigator. Each domain was rated as “low risk of bias,” “unclear risk of bias,” or “high risk of bias,” following the ROB 1.0 criteria. Summary risk-of-bias tables and graphs were generated using Review Manager (RevMan) version 5.4 to visually present the overall quality of the included studies.

### Statistical analysis

Statistical analyses were performed using Review Manager (RevMan) version 5.4. For time-to-event outcomes, HRs with 95% confidence intervals (CIs) reported in each study were extracted for pooled analysis. For dichotomous outcomes, risk ratios (RRs) with 95% CIs were calculated. Between-study heterogeneity was assessed using the I² statistic and the χ² test, with I² > 50% or P < 0.10 indicating substantial heterogeneity. Fixed-effect or random-effects models were selected based on the degree of heterogeneity. When high heterogeneity was present, sensitivity analyses were conducted to explore potential sources. Subgroup analyses were pre-specified according to disease stage (Stage II *vs*. Stage III) and chemotherapy regimen (FOLFOX *vs*. CAPOX).

To evaluate the certainty of evidence, the GRADE approach was applied, rating the quality of the main outcomes across five domains: risk of bias, inconsistency, indirectness, imprecision, and publication bias (20). TSA was performed using TSA version 0.9 to examine the robustness of cumulative evidence and determine whether the required information size (RIS) had been reached. TSA was conducted with a two-sided α = 0.05, power of 80%, and adjusted monitoring boundaries based on accrued data. Relative risk reduction (RRR) was specified for each outcome to calculate the RIS and to assess whether cumulative evidence crossed the predefined statistical thresholds (21).

## Results

### Study selection

**Figure 1:**
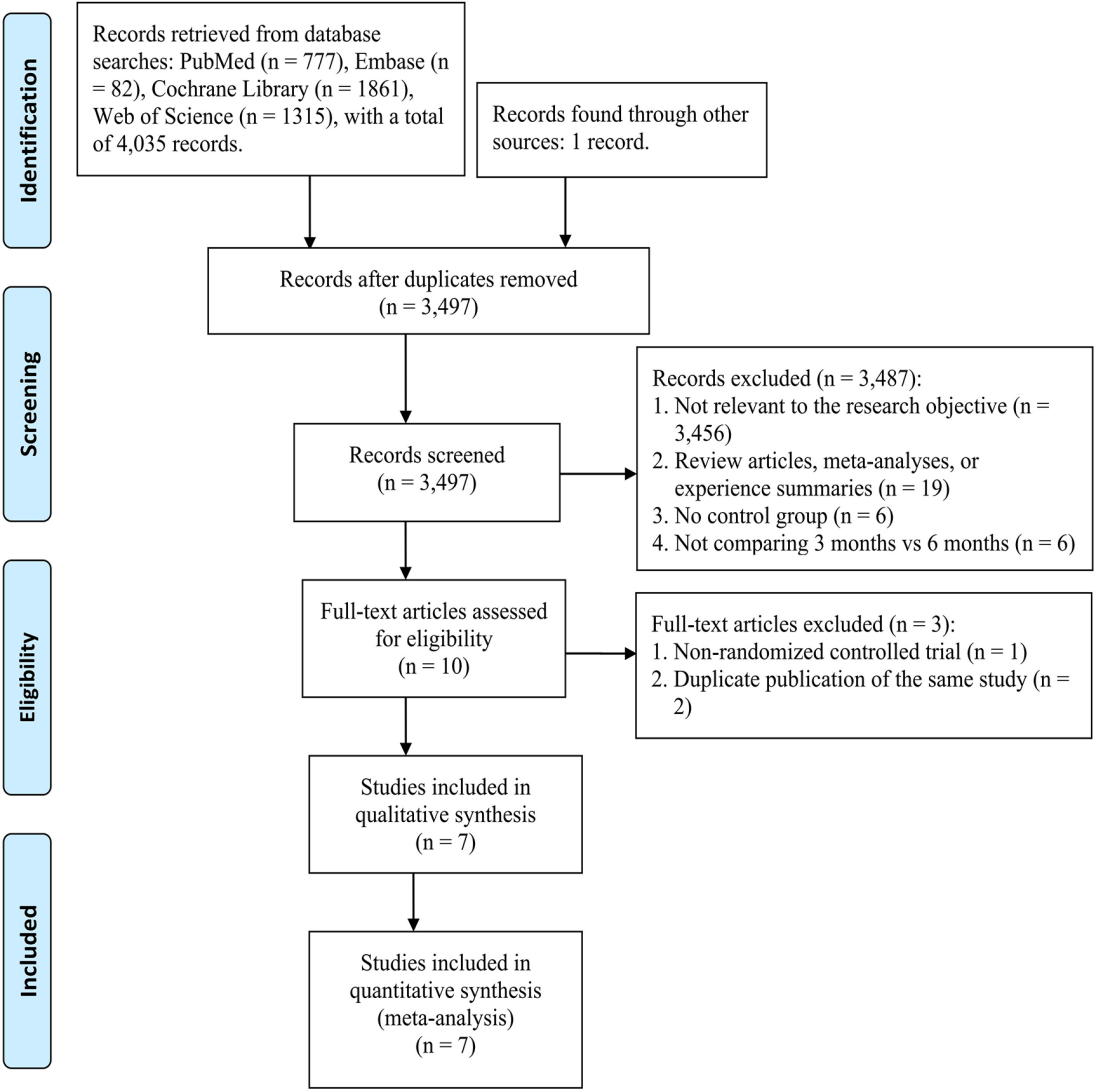
PRISMA flow diagram of the study selection process.

A total of 4,035 records were retrieved through database searching, and one additional record was identified through manual searching. After removing duplicates, 3,497 records remained for initial screening. Following title and abstract screening, 3,487 irrelevant studies were excluded, and 10 articles were assessed in full text. Of these, 3 studies were excluded (1 non-randomized study and 2 duplicate publications). Ultimately, 7 articles involving 6 RCTs (10–16), comprising a total of 16,420 patients, met the inclusion criteria and were included in the meta-analysis (**Figure 1**, **Table 1**). Of these, Yamazaki 2021 (15) and Yoshino 2019 (16) are part of the same ACHIEVE study, with Yamazaki 2021 (15) focusing on Stage II, and Yoshino 2019 (16) focusing on Stage III.

**Table 1:** Characteristics of the studies included in the meta-analysis.

Study	Name of trials	Country	Group	Sample size	Age (years)	Sex (Male/Female)	Median follow-up time	Chemotherapy	Tumor stage	Risk status
André 2018 (10)	IDEA France	France	3 months	1002	63.9 ± 9.4	563/439	4.3 years	FOLFOX6 (n=895), CAPOX (n= 107)	All Stage III	T4 and/or N2 (368)
			6 months	1008	63.9 ± 9.3	581/427	4.3 years	FOLFOX6 (n=914), CAPOX (n= 94)	All Stage III	T4 and/or N2 (396)
Iveson 2018 (11)	SCOT	UK, Denmark, Spain, Sweden, Australia,	3 months	3044	65(58–70)	1843/1201	37 months	FOLFOX (n=986), CAPOX (n= 2049)	Stage II (n=551), Stage III (n=2493)	T4 (917), N2 (754)
		and New Zealand	6 months	3044	65(58–70)	1844/1200	37 months	FOLFOX (n=988), CAPOX (n= 2056)	Stage II (n=545), Stage III (n=2499)	T4 (915), N2 (755)
Kim 2022 (12)	KCSG	Korean	3 months	893	58(52-65)	515/378	78.7 months	FOLFOX (n=620), CAPOX (n= 273)	Stage II (n=196), Stage III (n=697)	High-risk stageII (196), StageIII (T4 and/or N2) (221)
			6 months	895	58(52-65)	535/360	78.7 months	FOLFOX (n=617), CAPOX (n= 278)	Stage II (n=196), Stage III (n=699)	High-risk stageII (196), StageIII (T4 and/or N2) (243)
Petrelli 2021 (13)	TOSCA	Italy	3 months	1775	63.6 ± 9.4	1000/775	7 years	FOLFOX (n=1141), CAPOX (n= 634)	Stage II (n=618), Stage III (n=1144), Missing (n=13)	StageII—T4 (145), StageI II—high risk (396)
			6 months	1839	63.1 ± 9.8	1014/825	7 years	FOLFOX (n=1177), CAPOX (n= 662)	Stage II (n=631), Stage III (n=1196), Missing (n=12)	StageII—T4 (151), StageI II—high risk (408)
Souglako 2019 (14)	HORG-IDEA	Greece	3 months	557	67(20–81)	315/243	67 months	FOLFOX (n=195), CAPOX (n=362)	Stage II (n=206), Stage III (n=351)	T4 and/or N2 (200)
			6 months	558	65(22–82)	309/249	67 months	FOLFOX (n=196), CAPOX (n= 362)	Stage II (n=207), Stage III (n=351)	T4 and/or N2 (178)
Yamazaki 2021 (15)	ACHIEVE-2 trial	Japan	3 months	255	66(30-83)	140/115	36.1 months	FOLFOX (n=40), CAPOX (n= 215)	All Stage II	T4 (92), Number of harvested lymph nodes <12 (32)
			6 months	259	66(23-81)	152/107	36.1 months	FOLFOX (n=42), CAPOX (n= 217)	All Stage II	T4 (92), Number of harvested lymph nodes <12 (34)
Yoshino 2019 (16)	ACHIEVE	Japan	3 months	650	NA	329/321	39 months	FOLFOX (n=163), CAPOX (n=487)	All Stage III	T4 and/or N2 (351)
			6 months	641	NA	320/321	39 months	FOLFOX (n=159), CAPOX (n= 482)	All Stage III	T4 and/or N2 (344)

High-risk stage II patients were further analyzed based on the presence of T4, median number of nodes examined, tumour grade, and histologic subtypes. Similarly, stage III patients were categorized into high and low risk, with high risk defined by the presence of pT4 or pN2 disease. NA, not available; FOLFOX, fluorouracil, leucovorin, and oxaliplatin; CAPOX, capecitabine plus oxaliplatin.

### Quality assessment

**Figure 2:**
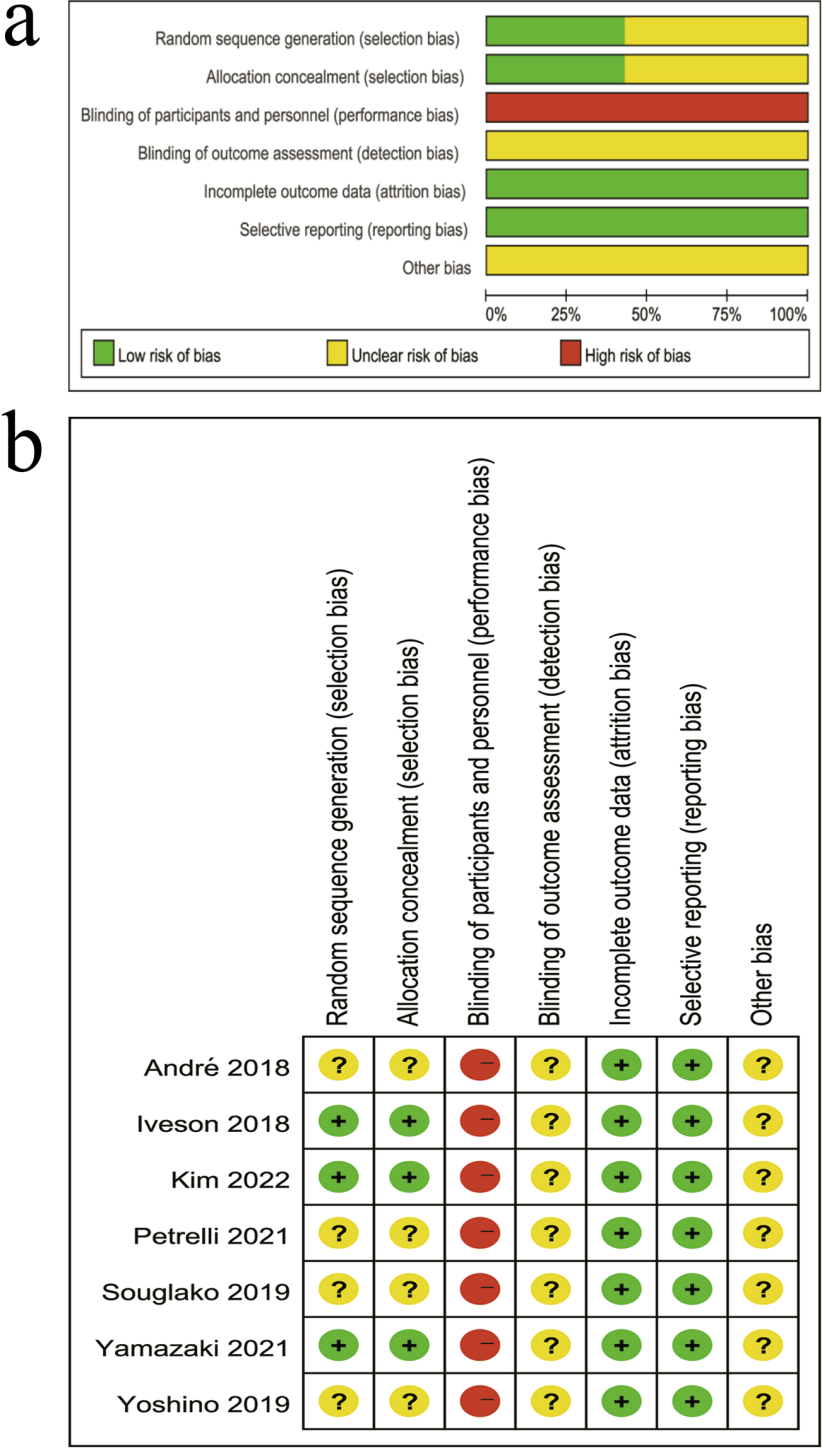
Risk of bias assessment for the included RCTs. **(a)** Summary of the risk of bias across all included studies. **(b)** Risk of bias assessment for each individual study across the seven ROB domains.

A total of three studies explicitly described the methods used for generating the random sequence (11, 12, 15), and two of them also reported the specific procedures for allocation concealment (11, 15). None of the included trials implemented blinding of participants or personnel, nor did they report whether outcome assessors were blinded. In addition, most studies lacked detailed descriptions of potential sources of bias. All trials provided complete or acceptable follow-up data, and no obvious selective reporting bias was identified (**Figure 2**).

### Efficacy outcomes

#### Primary outcomes in the oxaliplatin-based chemotherapy patients (unstratified by regimen)

**Figure 3:**
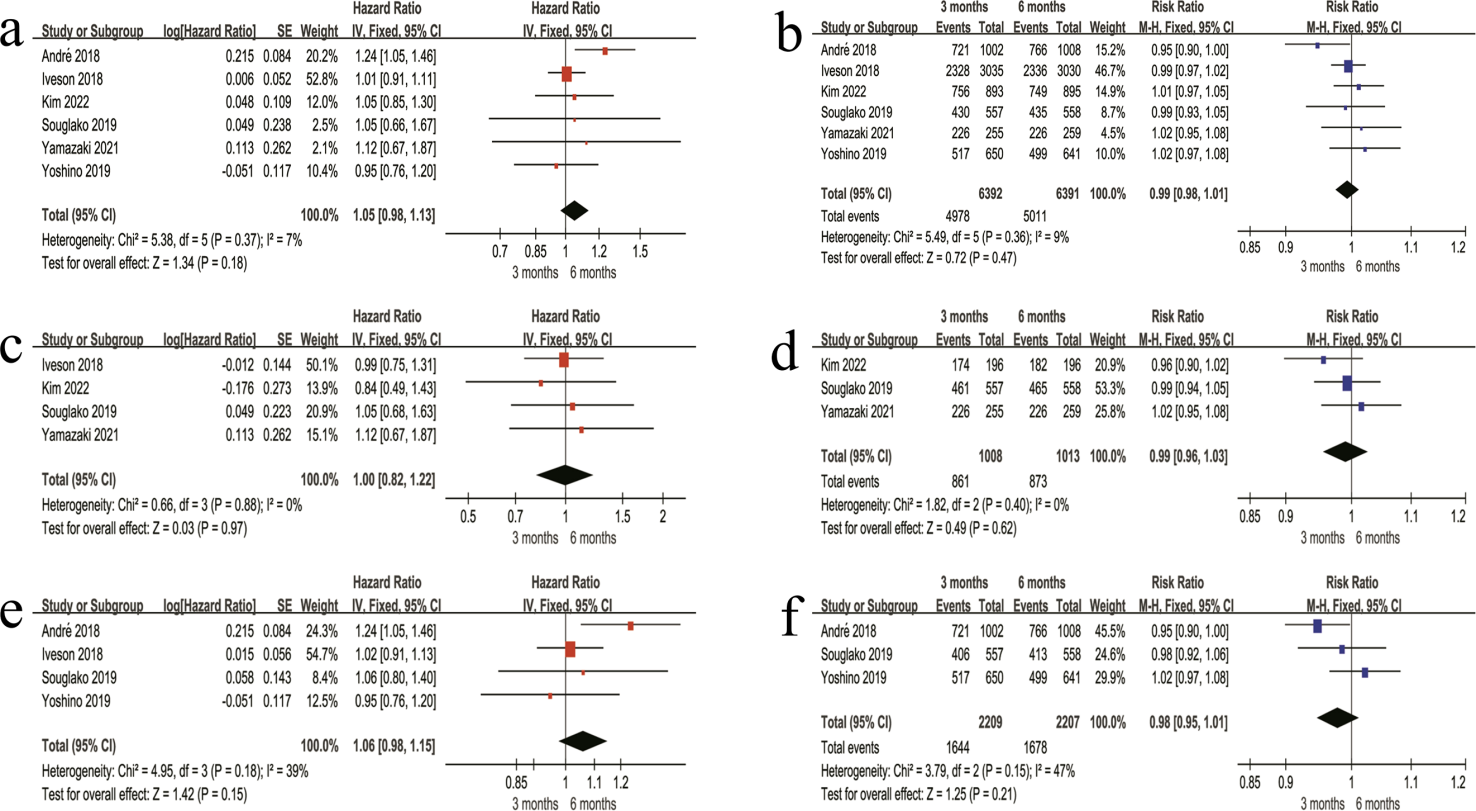
Forest plots of DFS outcomes in patients receiving oxaliplatin-based adjuvant chemotherapy. **(a)** Overall HR for DFS in the combined stage II + III patients. **(b)** Overall 3-year DFS rate in the combined stage II + III patients. **(c)** HR for DFS in stage II patients. **(d)** 3-year DFS rate in stage II patients. **(e)** HR for DFS in stage III patients. **(f)** 3-year DFS rate in stage III patients.

(1) In the overall stage II + III patients, the 3-month regimen showed a higher risk of DFS events compared with the 6-month regimen (HR = 1.05, 95% CI 0.98–1.13, P = 0.18) (**Figure 3a**). The 3-year DFS rate was 77.9% in the 3-month regimen and 78.4% in the 6-month regimen, with no significant difference between the two regimens (RR = 0.99, 95% CI 0.98–1.01, P = 0.47) (**Figure 3b**). (2) Among stage II patients, DFS did not differ significantly between the two regimens (HR = 1.00, 95% CI 0.82–1.22, P = 0.97) (**Figure 3c**). Likewise, the 3-year DFS rate was similar between regimens (85.4% *vs* 86.2%; RR = 0.99, 95% CI 0.96–1.03, P = 0.62) (**Figure 3d**). (3) For stage III patients, DFS also showed no significant difference (HR = 1.06, 95% CI 0.98–1.15, P = 0.15) (**Figure 3e**). The 3-year DFS rate was 74.2% in the 3 months regimen and 76.0% in the 6 months regimen, without a statistically significant difference (RR = 0.98, 95% CI 0.95–1.01, P = 0.21) (**Figure 3f**).

#### High-risk stage II patients analysis (unstratified by regimen)

**Figure 4:**
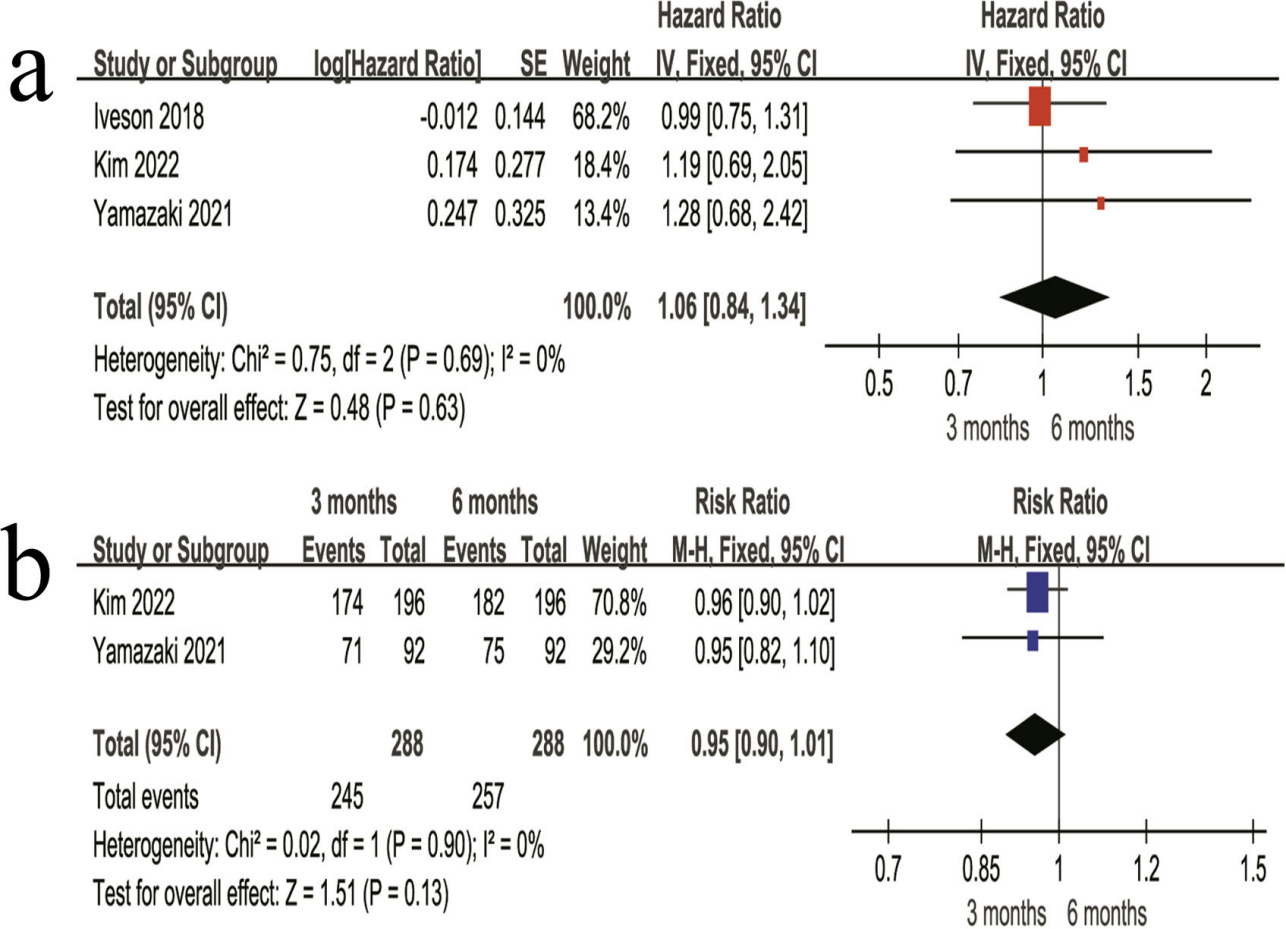
Forest plots of DFS outcomes in high-risk stage II patients. **(a)** HR for DFS comparing the 3-month and 6-month regimens in high-risk stage II patients. **(b)** 3-year DFS rate comparing the 3-month and 6-month regimens in high-risk stage II patients.

Among high-risk stage II patients, no significant difference in DFS was observed between the 3-month and 6-month regimens (HR = 1.06, 95% CI 0.84–1.34, P = 0.63) (**Figure 4a**). The 3-year DFS rate was comparable between the two regimens, with rates of 85.1% in the 3-month regimen and 89.2% in the 6-month regimen, and no statistically significant difference was observed (RR = 0.95, 95% CI 0.90–1.01, P = 0.13) (**Figure 4b**).

#### DFS and 3-year DFS rate in the FOLFOX regimen

**Figure 5:**
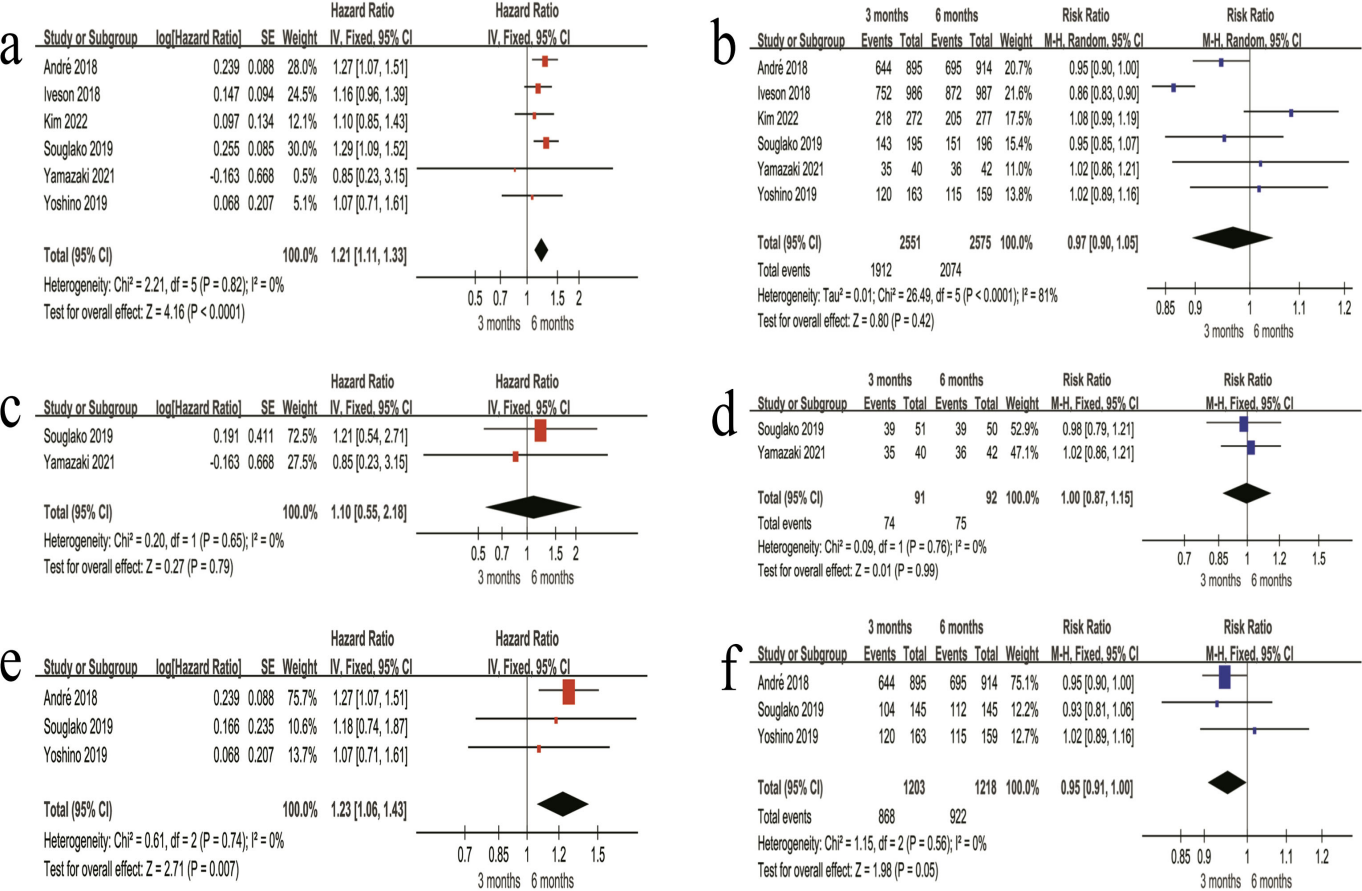
Forest plots of DFS outcomes for patients treated with the FOLFOX adjuvant chemotherapy regimen. **(a)** Overall HR for DFS in the combined stage II + III patients receiving FOLFOX. **(b)** Overall 3-year DFS rate in the combined stage II + III patients receiving FOLFOX. **(c)** HR for DFS in stage II patients receiving FOLFOX. **(d)** 3-year DFS rate in stage II patients receiving FOLFOX. **(e)** HR for DFS in stage III patients receiving FOLFOX. **(f)** 3-year DFS rate in stage III patients receiving FOLFOX.

(1) In the combined stage II + III patients, the 3-month regimen was associated with a higher DFS risk compared with the 6-month regimen (HR = 1.21, 95% CI 1.11–1.33, P < 0.001) (**Figure 5a**). The 3-year DFS rate was 74.9% in the 3-month regimen and 80.5% in the 6-month regimen, with no significant difference between the two regimens (RR = 0.97, 95% CI 0.90–1.05, P = 0.42) (**Figure 5b**). (2) Among stage II patients, DFS did not differ significantly between the two regimens (HR = 1.10, 95% CI 0.55–2.18, P = 0.79) (**Figure 5c**). The 3-year DFS rate was also similar between regimens (81.3% *vs* 81.5%; RR = 1.00, 95% CI 0.87–1.15, P = 0.99) (**Figure 5d**). (3) In stage III patients, pooled DFS results indicated a higher risk in the 3-month regimen (HR = 1.23, 95% CI 1.06–1.43, P = 0.007) (**Figure 5e**). The 3-year DFS rate was 72.2% in the 3-month regimen and 75.7% in the 6-month regimen, with no statistically significant difference (RR = 0.95, 95% CI 0.91–1.00, P = 0.05) (**Figure 5f**).

#### DFS and 3-year DFS rate in the CAPOX regimen

**Figure 6:**
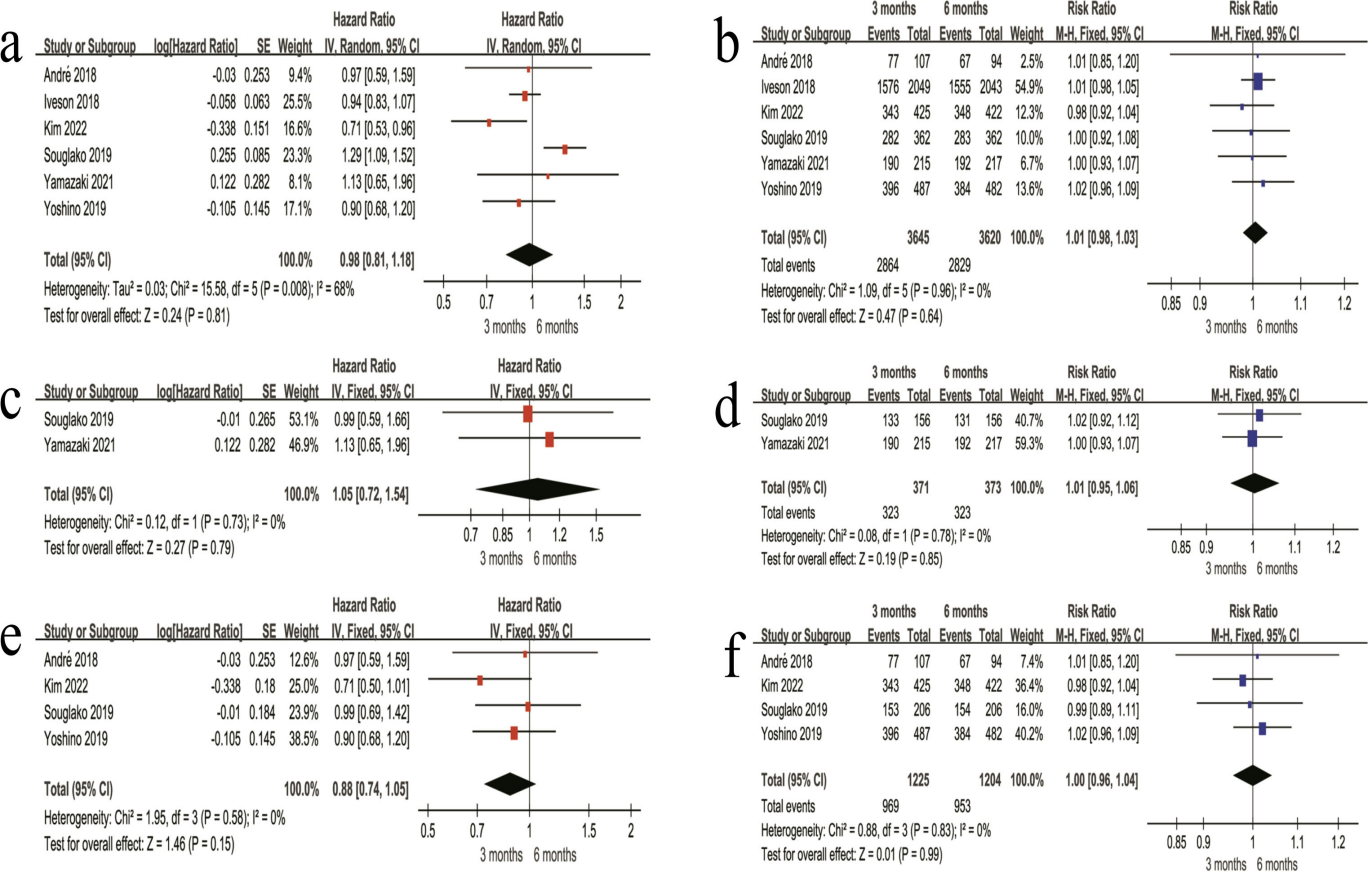
Forest plots of DFS outcomes for patients treated with the CAPOX regimen. **(a)** Overall HR for DFS in the combined stage II + III patients receiving CAPOX. **(b)** Overall 3-year DFS rate in the combined stage II + III patients receiving CAPOX. **(c)** HR for DFS in stage II patients receiving CAPOX. **(d)** 3-year DFS rate in stage II patients receiving CAPOX. **(e)** HR for DFS in stage III patients receiving CAPOX. **(f)** 3-year DFS rate in stage III patients receiving CAPOX.

(1) In the combined stage II + III patients receiving the CAPOX regimen, pooled DFS analysis showed no significant difference between the 3-month and 6-month regimens (HR = 0.98, 95% CI 0.81–1.18, P = 0.81) (**Figure 6a**). The 3-year DFS rate was 78.6% in the 3-month regimen and 78.1% in the 6-month regimen, with no significant difference between the two regimens (RR = 1.01, 95% CI 0.98–1.03, P = 0.64) (**Figure 6b**). (2) Among stage II patients treated with CAPOX, DFS did not differ significantly between the 3-month and 6-month regimens (HR = 1.05, 95% CI 0.72–1.54, P = 0.79) (**Figure 6c**). The 3-year DFS rate was also comparable between regimens (87.1% *vs* 86.6%; RR = 1.01, 95% CI 0.95–1.06, P = 0.85) (**Figure 6d**). (3) In stage III patients receiving CAPOX, there was no significant difference in DFS between the 3-month and 6-month regimens (HR = 0.88, 95% CI 0.74–1.05, P = 0.15) (**Figure 6e**). The 3-year DFS rate was identical in both regimens (79.1% *vs* 79.1%; RR = 1.00, 95% CI 0.96–1.04, P = 0.99) (**Figure 6f**).

#### OS and 3-year OS rate

**Figure 7:**
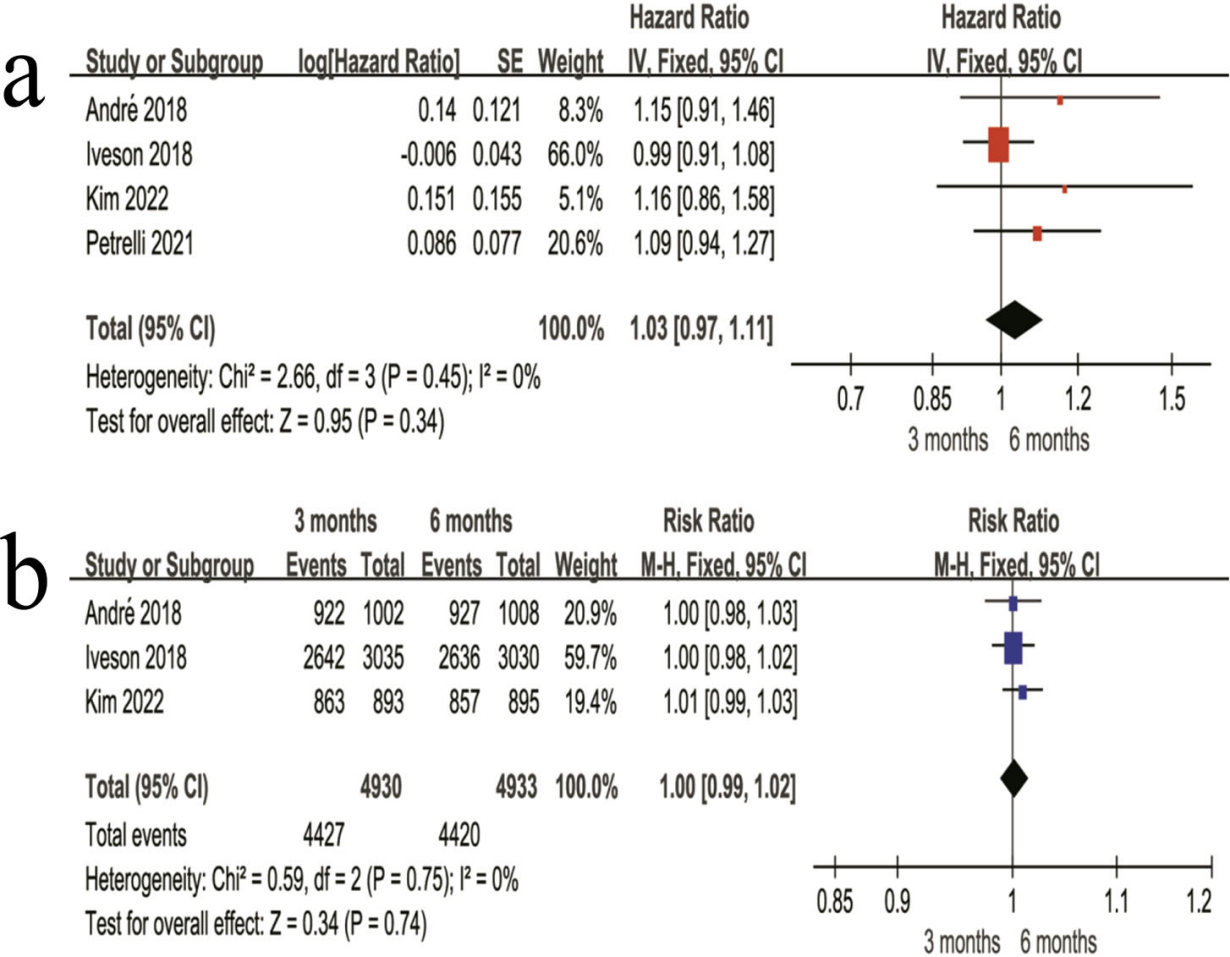
Forest plots of OS outcomes in the combined stage II + III patients. **(a)** Overall HR for OS in the combined stage II + III patients. **(b)** Overall 3-year OS rate in the combined stage II + III patients.

In the combined stage II + III patients, OS did not differ significantly between the 3-month and 6-month regimens (HR = 1.03, 95% CI 0.97–1.11, P = 0.34) (**Figure 7a**). The 3-year OS rate was 89.8% in the 3-month regimen and 89.6% in the 6-month regimen, with no significant difference between the two regimens (RR = 1.00, 95% CI 0.99–1.02, P = 0.74) (**Figure 7b**).

### Safety and tolerability outcomes

#### Chemotherapy completion rate

**Figure 8:**
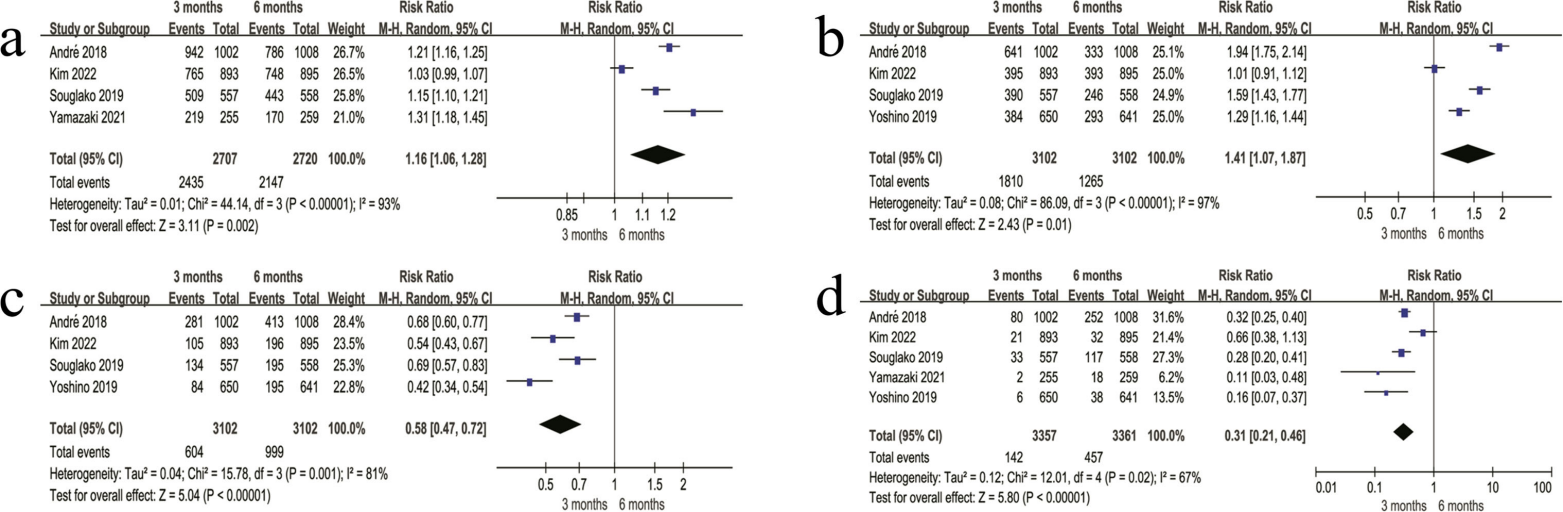
Forest plots of chemotherapy completion rate and PSN outcomes. **(a)** Chemotherapy completion rate in the 3-month vs. 6-month regimens. **(b)** Grade 1 PSN in the 3-month vs. 6-month regimens. **(c)** Grade 2 PSN in the 3-month vs. 6-month regimens. **(d)** Grade 3–4 PSN in the 3-month vs. 6-month regimens.

The chemotherapy completion rate was higher in the 3-month regimen than in the 6-month regimen (89.9% *vs* 78.9%), and pooled analysis showed a significant difference between the two regimens (RR = 1.16, 95% CI 1.06–1.28, P = 0.002) (**Figure 8a**).

#### PSN

For grade 1 PSN, the incidence was higher in the 3-month regimen (58.3% *vs* 40.7%), with a significant difference between regimens (RR = 1.41, 95% CI 1.07–1.87, P = 0.01) (**Figure 8b**). For grade 2 PSN, the 3-month regimen had a lower incidence (19.4% *vs* 32.2%), and the pooled RR indicated a significant difference (RR = 0.58, 95% CI 0.47–0.72, P < 0.00001) (**Figure 8c**). For grade 3–4 PSN, the incidence was markedly lower in the 3-month regimen (4.2% *vs* 13.6%), and the difference between regimens was statistically significant (RR = 0.31, 95% CI 0.21–0.46, P < 0.00001) (**Figure 8d**).

### Sensitivity analysis

To assess the robustness of the findings, leave-one-out sensitivity analyses were performed for outcomes with relatively high heterogeneity, including the overall 3-year DFS rate in the combined stage II + III patients (FOLFOX regimen), overall HR for DFS in the combined stage II + III patients (CAPOX regimen), chemotherapy completion rate, grade 1 PSN, grade 2 PSN, and grade 3–4 PSN. The results showed that sequential exclusion of any single study did not materially alter the pooled effect estimates, indicating that the overall conclusions remained stable.

### GRADE evidence summary

Most efficacy outcomes were rated as moderate-certainty evidence, except for the following, which were rated as low-certainty: Overall 3-year DFS rate in the combined stage II + III patients (FOLFOX regimen), HR for DFS in stage II patients (FOLFOX regimen), Overall HR for DFS in the combined stage II + III patients (CAPOX regimen), HR for DFS in stage II patients (CAPOX regimen), chemotherapy completion rate, grade 1 PSN, grade 2 PSN, and grade 3–4 PSN (**Supplementary Table 1**).

### TSA results

TSA showed that, except for the 3-year DFS rate in stage II patients (FOLFOX6 regimen) and all grades of PSN which did not reach the RIS, all other outcome measures reached the RIS (**Supplementary Figures 1**–**15**).

## Discussion

Adjuvant chemotherapy is a key strategy to reduce recurrence and improve survival in patients with CRC after curative resection (22). Evidence indicates that patients with early- or intermediate-stage CRC treated with surgery alone face a 5-year recurrence risk as high as 40–50% (23). Therefore, the addition of fluoropyrimidine- and platinum-based adjuvant chemotherapy significantly reduces recurrence and improves survival outcomes (24). This study systematically analyzed six large RCTs, encompassing a total of 16,420 patients with stage II and III CRC who received oxaliplatin-based adjuvant chemotherapy (FOLFOX or CAPOX). It represents one of the most comprehensive and updated evidence syntheses comparing different treatment durations (3 months *vs* 6 months). In the overall stage II + III patients, the 3-month regimen showed no difference in DFS HR compared to the 6-month regimen, with a statistically insignificant result. The absolute difference in 3-year DFS rate between the two regimens was only 0.5 percentage points, indicating minimal clinical relevance and aligning closely with the findings of the international IDEA collaboration (25). In the stage-stratified analyses, no significant difference in DFS or 3-year DFS rate was observed between treatment durations among stage II and high-risk stage II patients, consistent with the TOSCA trial findings in high-risk stage II disease. These results suggest that shortening treatment to 3 months does not compromise outcomes in this subgroup (26). Among stage III patients, both DFS and the 3-year DFS rate were slightly lower in the 3-month group compared with the 6-month group; however, the differences did not reach statistical significance. It is worth noting that these results are not entirely consistent with the IDEA pooled analysis, in which the 3-month regimen did not meet the predefined non-inferiority criteria in the overall stage III patients (25).

Since the MOSAIC trial first demonstrated the significant survival benefits of the FOLFOX regimen in the adjuvant treatment of stage III colorectal cancer, FOLFOX has become one of the global standard adjuvant chemotherapy options (24). In the present study, results from the FOLFOX subgroup showed that, in the overall stage II + III patients, the 3-month regimen was associated with a significantly higher DFS risk compared with the 6-month regimen, suggesting that shortening treatment duration may compromise disease control. Although the difference in 3-year DFS rates did not reach statistical significance, the trend favored improved long-term outcomes with extended therapy. Among stage II patients, no significant differences in DFS or 3-year DFS rate were observed between the 3-month and 6-month FOLFOX regimens, indicating that treatment duration may have limited impact on prognosis in this subgroup. In contrast, the differences were more pronounced in stage III patients: the 3-month regimen resulted in a significantly higher DFS risk and a slightly lower 3-year DFS rate compared with the 6-month regimen. These findings suggest that, under the FOLFOX regimen, stage III colorectal cancer patients may derive greater benefit from extending adjuvant chemotherapy to 6 months.

The CAPOX regimen, consisting of oral fluoropyrimidine prodrug capecitabine combined with oxaliplatin, is one of the major standard adjuvant chemotherapy options for colorectal cancer (27). In the present study, results from the CAPOX subgroup showed no significant difference in DFS between the 3-month and 6-month regimens in the overall stage II + III patients, and the 3-year DFS rates were likewise comparable. In both the stage II and stage III subgroup analyses, no significant differences were observed between treatment durations. This contrasts with the results in the stage III patients under the FOLFOX regimen, where the longer treatment duration showed more benefit. This difference may be related to the characteristics of the regimen: the oral component of CAPOX may have improved the completion rate and tolerance of the longer treatment duration, thereby diminishing the additional benefits of extending the treatment. These findings suggest that, under the CAPOX regimen, shortening the treatment duration to 3 months does not compromise overall therapeutic efficacy. In the overall stage II + III patients, no significant differences were found in OS between the 3-month and 6-month oxaliplatin-based regimens, and the 3-year OS rates were nearly identical. This indicates that shortening the duration of adjuvant chemotherapy does not diminish long-term overall survival benefits. Although the difference in OS between the two groups did not reach statistical significance, this finding of survival equivalence holds important clinical significance. Short-course chemotherapy can achieve survival outcomes similar to those of the standard regimen by reducing adverse reactions, decreasing treatment-related toxicity, and improving patient adherence to treatment. Therefore, short-course treatment regimens offer an effective clinical strategy for balancing survival benefits with quality of life. These observations are consistent with the results of multiple large international trials and pooled analyses. The final report of the IDEA collaboration showed that the difference in 5-year OS between the 3-month and 6-month regimens was only 0.4% and did not reach statistical significance (28). This suggests that although short-course therapy may show slight differences in DFS, such differences do not translate into meaningful long-term survival disadvantages.

The 3-month oxaliplatin-based adjuvant chemotherapy regimen demonstrated a significantly higher treatment completion rate compared with the 6-month regimen, indicating that shortening the treatment duration can effectively improve patient adherence and treatment accessibility. Because the primary dose-limiting toxicity of oxaliplatin is cumulative PSN, the length of therapy is closely associated with the severity of neurotoxicity (29). Our findings further revealed that the incidences of grade 2 and grade 3–4 PSN were all significantly lower in the 3-month regimen than in the 6-month regimen, with severe (grade 3–4) PSN occurring in only 4.2% of patients receiving 3 months of therapy compared with 13.6% in those receiving 6 months— a highly significant difference. The incidence of grade 1 PSN was higher in the 3-month regimen compared to the 6-month regimen, reflecting that fewer patients progressed to grade 2–4 PSN, resulting in more patients remaining at grade 1.Peripheral neurotoxicity not only impairs quality of life (QoL) but also negatively impacts long-term survivorship, as it can lead to treatment interruptions or dose reductions, which in turn decrease patient adherence and the effectiveness of treatment (30, 31). Thus, by reducing toxicity and improving completion rates, shorter regimens may enhance the “effective dose delivery” and ultimately contribute to better overall patient outcomes, including preserving long-term survival benefits and improving QoL. It should be noted, however, that the number of included studies in the present analysis was relatively limited, and independent subgroup analyses for different chemotherapy regimens (FOLFOX *vs* CAPOX) were not performed. Given the pharmacokinetic and toxicity profile differences between these two regimens, some degree of heterogeneity may exist. Future prospective studies with larger sample sizes are needed to further elucidate regimen-specific toxicity patterns and adherence outcomes across different treatment durations.

According to the GRADE evidence quality assessment, the majority of the primary outcomes in this study are rated as moderate-quality evidence, indicating that the results are reasonably reliable, but should be interpreted with caution. Moderate-quality evidence suggests that, while the study results support the effectiveness of the current treatment regimen, there remains a certain degree of uncertainty, particularly due to heterogeneity across studies and potential sources of bias, which may affect the robustness of the conclusions. On the other hand, some subgroup evidence was rated as low quality, primarily due to insufficient reporting of random sequence generation and allocation concealment, high heterogeneity between studies, and wide confidence intervals for effect sizes, which contribute to greater uncertainty in the results. The TSA results further demonstrated that most key outcomes—including the overall 3-year DFS in the combined stage II + III patients, the 3-year DFS in stage III patients, and the overall DFS and OS outcomes under both the FOLFOX and CAPOX regimens—had already reached the RIS and crossed the sequential monitoring boundaries. These findings suggest that the current cumulative evidence is adequate and supports the robustness of the conclusions.

This study has several limitations that should be taken into consideration when interpreting the findings. First, the number of included studies was limited, and sample distributions were uneven. Although multiple RCTs were pooled, some subgroup sample sizes were relatively small, resulting in inadequate statistical power. The lack of blinding in most of the included studies may have introduced bias, affecting the internal validity of the findings. Variations in follow-up duration and endpoint definitions across studies may also have influenced the pooled effect estimates. Some included trials enrolled both colon and rectal cancer patients, which may introduce potential heterogeneity and affect the generalizability of the results. Second, the considerable heterogeneity among the included studies, indicated by the I² statistic, suggests that the results may not be entirely consistent across different populations and treatment regimens. Third, the certainty of evidence for some outcomes was low. According to the GRADE assessment, several subgroup outcomes were rated as low-certainty evidence, indicating that these conclusions should be interpreted with caution. Fourth, the risk of bias assessment using ROB 1.0 revealed potential limitations in the methodological quality of the included trials, particularly in the areas of random sequence generation and allocation concealment. This study used the Cochrane Risk of Bias Assessment Tool version 1.0 for risk of bias evaluation. Although this tool has been widely used, the current Cochrane standard recommends the updated version 2.0. This methodological choice may affect the assessment of the internal validity of the included studies and should be considered a potential limitation of this systematic review. Fifth, due to insufficient numbers of eligible studies, we could not further explore differences in toxicity or adherence between treatment durations within individual chemotherapy regimens (FOLFOX *vs* CAPOX), which may underestimate regimen-dependent effects. Sixth, this analysis captures acute PSN grading, but chronic or persistent neuropathy years after treatment, which is a major quality-of-life concern, was not addressed. Seventh, publication bias could not be assessed due to fewer than 10 studies, which should be considered as a limitation. Future research should validate these findings through larger, multicenter prospective studies.

## Conclusion

For stage II patients, regardless of whether FOLFOX or CAPOX is used, there is no significant difference between 3-month regimen and 6-month regimen in terms of DFS or 3-year DFS rate. Given the lower toxicity and higher completion rate, a 3-month regimen can be preferentially considered. For stage III patients, the effectiveness of the two treatment durations is comparable under the CAPOX regimen; however, within the FOLFOX regimen, 3-month regimen appears to be somewhat inferior to 6-month regimen in DFS, suggesting that stage III patients receiving FOLFOX may still benefit from a 6-month regimen.

## Data Availability

All data used in this study were extracted from previously published articles and are fully accessible in the public domain. No restrictions apply to the dataset. Requests to access these datasets should be directed to HW, zlys135475@163.com.

## Supplementary material

Supplementary Figure 1 TSA results for overall 3-year DFS rate (Stage II + III). Note: RIS boundaries are not shown due to the first study exceeding 100% of the information fraction (RIS = 1067). Relative risk reduction (RRR) is set to 10%.

Supplementary Figure 2 TSA results for 3-year DFS rate in stage II patients. Note: RIS boundaries are not shown due to the first study exceeding 100% of the information fraction (RIS = 627). RRR is set to 10%.

Supplementary Figure 3 TSA results for 3-year DFS rate in stage III patients. Note: The cumulative value exceeds the RIS boundary (RIS = 2058). RRR is set to 10%.

Supplementary Figure 4 TSA results for 3-year DFS rate in high-risk stage II patients. Note: The cumulative value exceeds the RIS boundary (RIS = 511). RRR is set to 10%.

Supplementary Figure 5 TSA results for overall 3-year DFS rate (stage II + III, FOLFOX regimen). Note: The cumulative value exceeds the RIS boundary (RIS = 4589). RRR is set to 10%.

Supplementary Figure 6 TSA results for 3-year DFS rate in stage II patients (FOLFOX regimen). Note: The cumulative value does not exceed the RIS boundary (RIS = 827). RRR is set to 10%.

Supplementary Figure 7 TSA results for 3-year DFS rate in stage III patients (FOLFOX regimen). Note: RIS boundaries are not shown due to the first study exceeding 100% of the information fraction (RIS = 1107). RRR is set to 10%.

Supplementary Figure 8 TSA results for overall 3-year DFS rate (Stage II + III, CAPOX regimen). Note: The cumulative value exceeds the RIS boundary (RIS = 986). RRR is set to 10%.

Supplementary Figure 9 TSA results for 3-year DFS rate in stage II patients (CAPOX regimen). Note: The cumulative value exceeds the RIS boundary (RIS = 611). RRR is set to 10%.

Supplementary Figure 10 TSA results for 3-year DFS rate in stage III patients (CAPOX regimen). Note: The cumulative value exceeds the RIS boundary (RIS = 938). RRR is set to 10%.

Supplementary Figure 11 TSA results for overall 3-year OS rate (stage II + III). Note: The first study exceeds the RIS boundary (RIS = 496). RRR is set to 10%.

Supplementary Figure 12 TSA results for chemotherapy completion rate. Note: The cumulative value exceeds the RIS boundary (RIS = 3714). RRR is set to 20%.

Supplementary Figure 13 TSA results for grade 1 PSN. Note: The cumulative value does not exceed the RIS boundary (RIS = 31609). RRR is set to 20%.

Supplementary Figure 14 TSA results for grade 2 PSN. Note: The cumulative value does not exceed the RIS boundary (RIS = 8205). RRR is set to 20%.

Supplementary Figure 15 TSA results for grade 3–4 PSN. Note: The cumulative value does not exceed the RIS boundary (RIS = 13815). RRR is set to 20%.
