# Supplementary figures and images for: Efficacy and safety of 3-month versus 6-month oxaliplatin-based adjuvant chemotherapy in colorectal cancer: a systematic review and meta-analysis
Source: Front Oncol. 2026 Feb 2;16:1762273. doi: 10.3389/fonc.2026.1762273 (PMC12907182; doi:10.3389/fonc.2026.1762273)

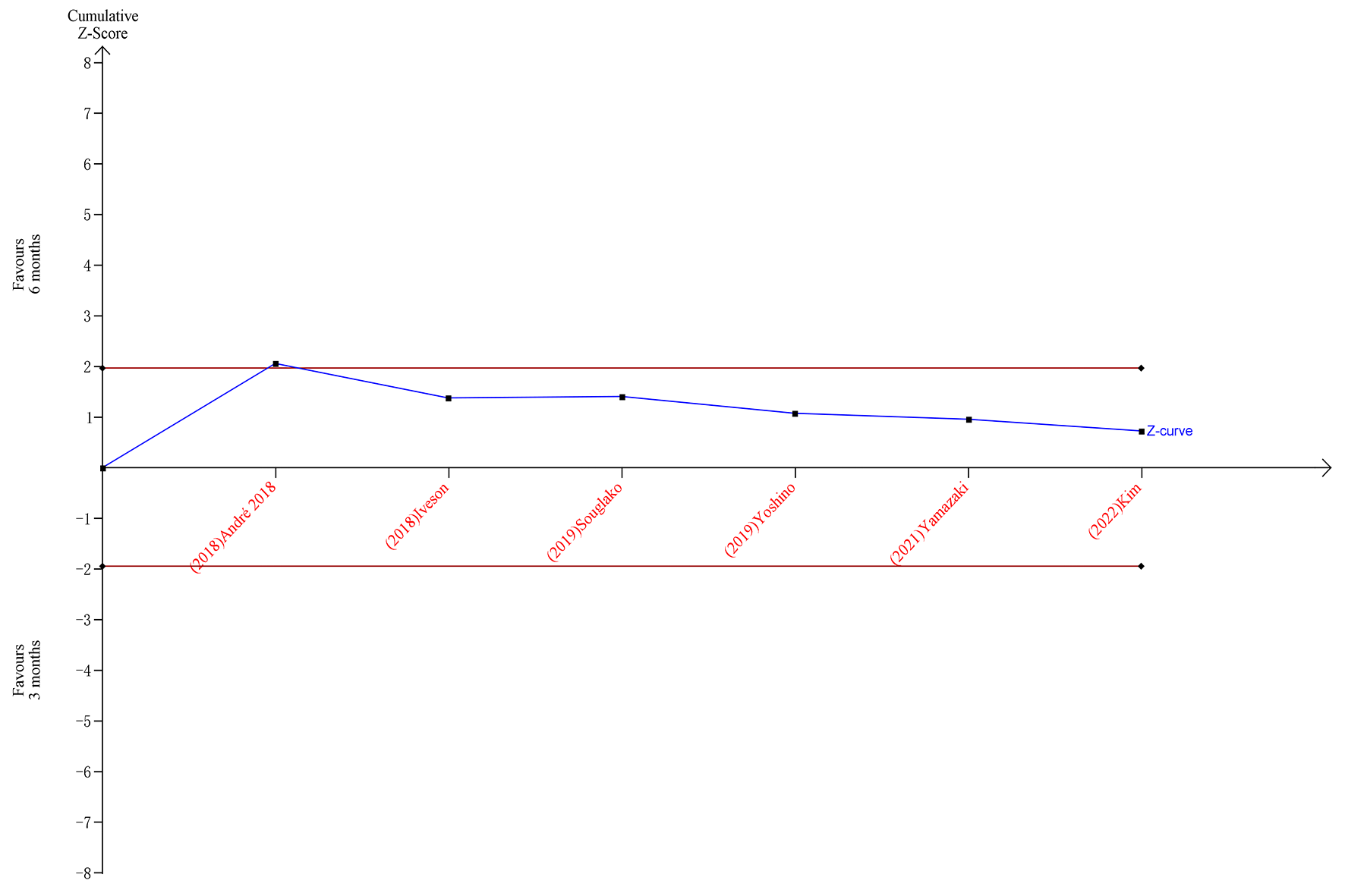

Supplement: Supplementary Figure 1 — TSA results for overall 3-year DFS rate (Stage II + III). Note: RIS boundaries are not shown due to the first study exceeding 100% of the information fraction (RIS = 1067). Relative risk reduction (RRR) is set to 10%. [file Image1.tif]

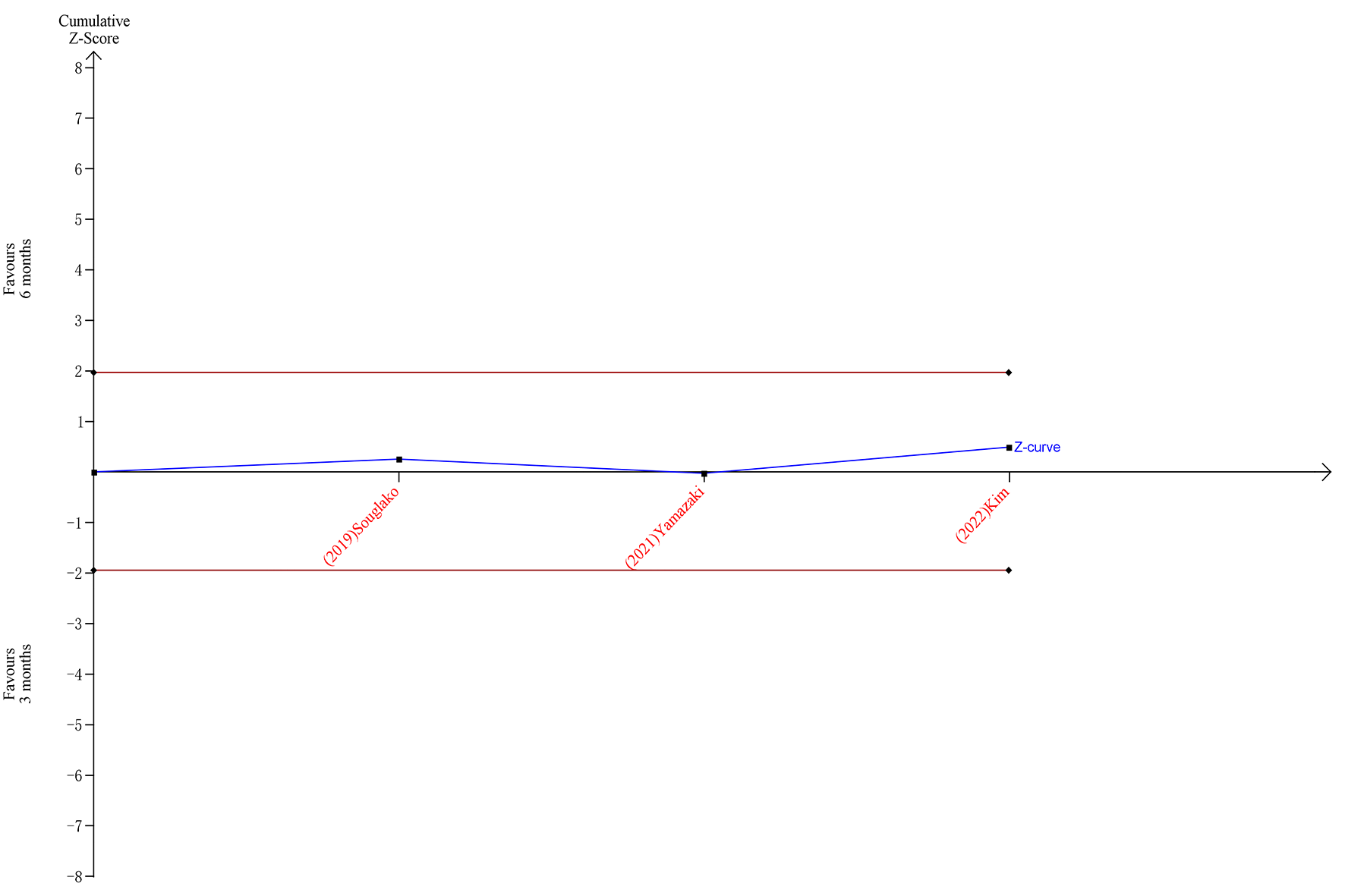

Supplement: Supplementary Figure 2 — TSA results for 3-year DFS rate in stage II patients. Note: RIS boundaries are not shown due to the first study exceeding 100% of the information fraction (RIS = 627). RRR is set to 10%. [file Image2.tif]

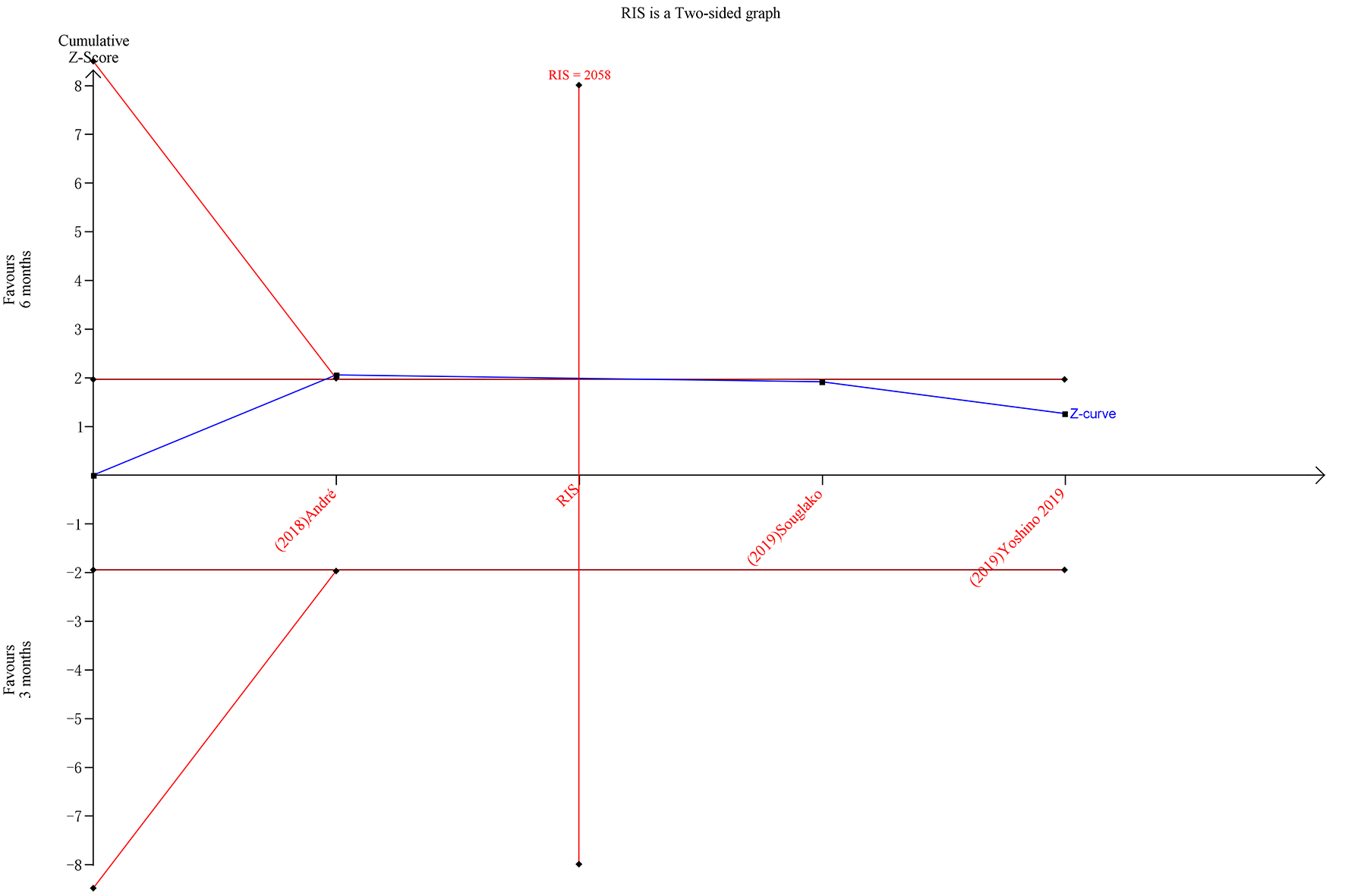

Supplement: Supplementary Figure 3 — TSA results for 3-year DFS rate in stage III patients. Note: The cumulative value exceeds the RIS boundary (RIS = 2058). RRR is set to 10%. [file Image3.tif]

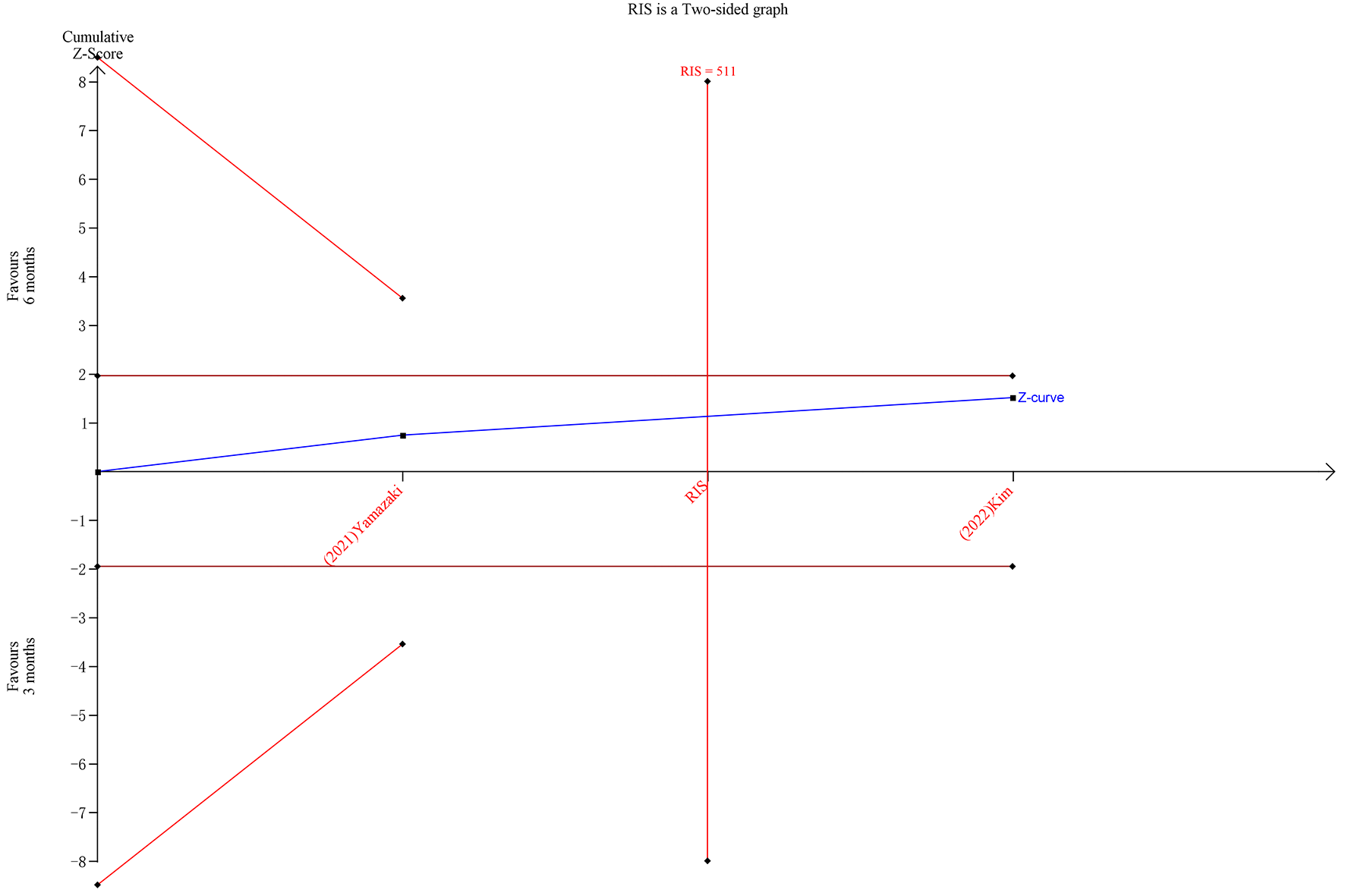

Supplement: Supplementary Figure 4 — TSA results for 3-year DFS rate in high-risk stage II patients. Note: The cumulative value exceeds the RIS boundary (RIS = 511). RRR is set to 10%. [file Image4.tif]

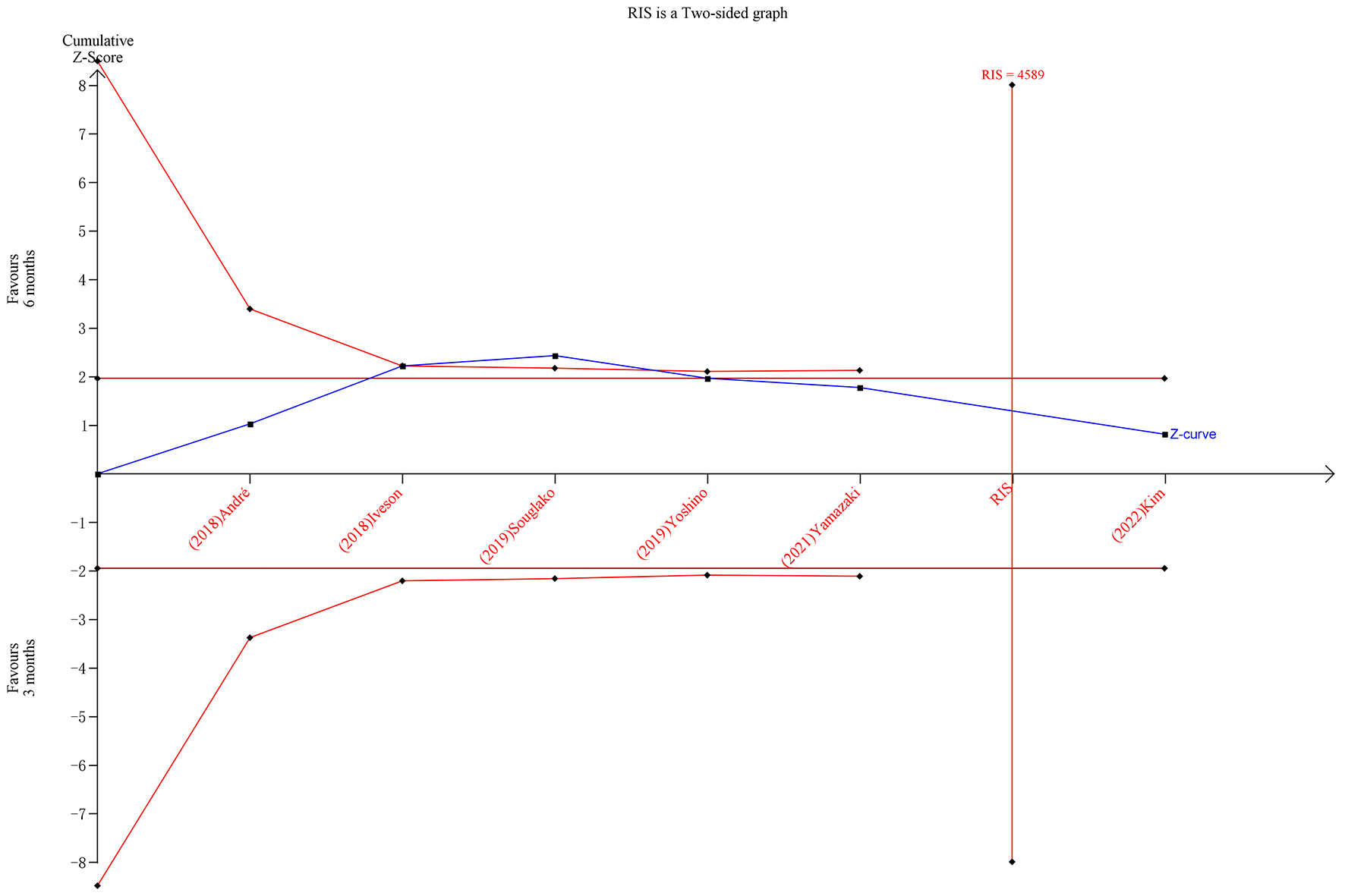

Supplement: Supplementary Figure 5 — TSA results for overall 3-year DFS rate (stage II + III, FOLFOX regimen). Note: The cumulative value exceeds the RIS boundary (RIS = 4589). RRR is set to 10%. [file Image5.tif]

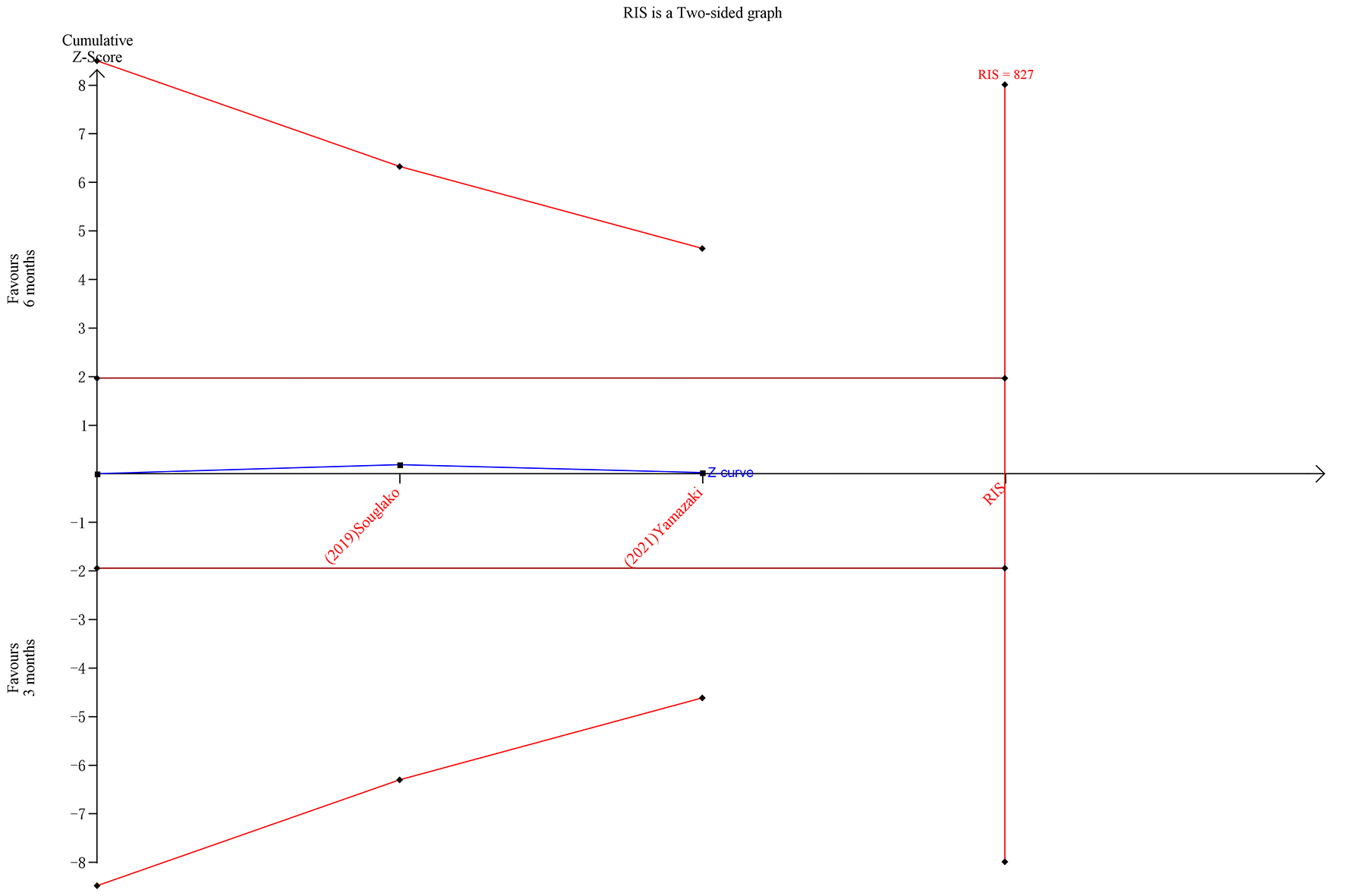

Supplement: Supplementary Figure 6 — TSA results for 3-year DFS rate in stage II patients (FOLFOX regimen). Note: The cumulative value does not exceed the RIS boundary (RIS = 827). RRR is set to 10%. [file Image6.tif]

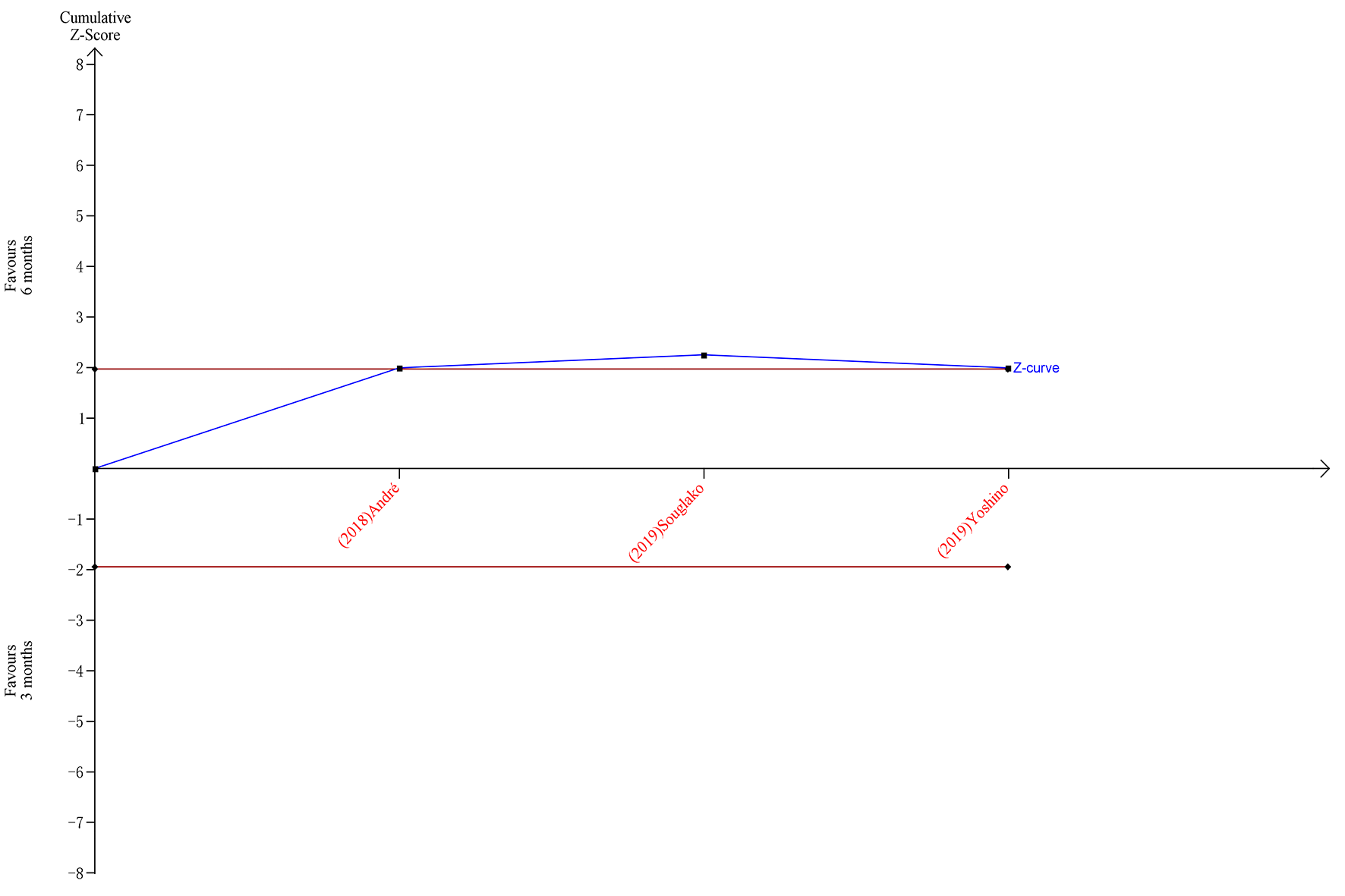

Supplement: Supplementary Figure 7 — TSA results for 3-year DFS rate in stage III patients (FOLFOX regimen). Note: RIS boundaries are not shown due to the first study exceeding 100% of the information fraction (RIS = 1107). RRR is set to 10%. [file Image7.tif]

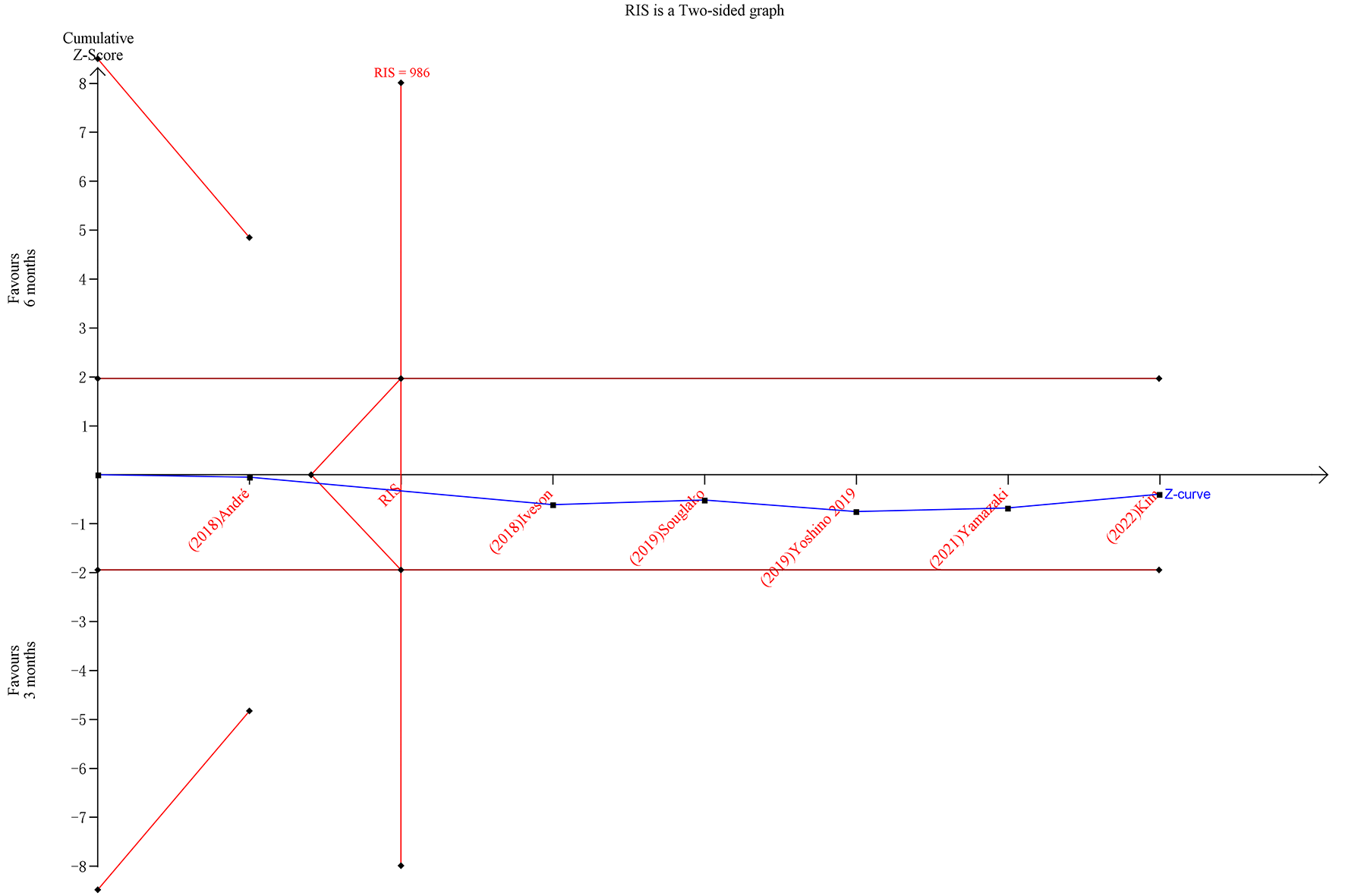

Supplement: Supplementary Figure 8 — TSA results for overall 3-year DFS rate (Stage II + III, CAPOX regimen). Note: The cumulative value exceeds the RIS boundary (RIS = 986). RRR is set to 10%. [file Image8.tif]

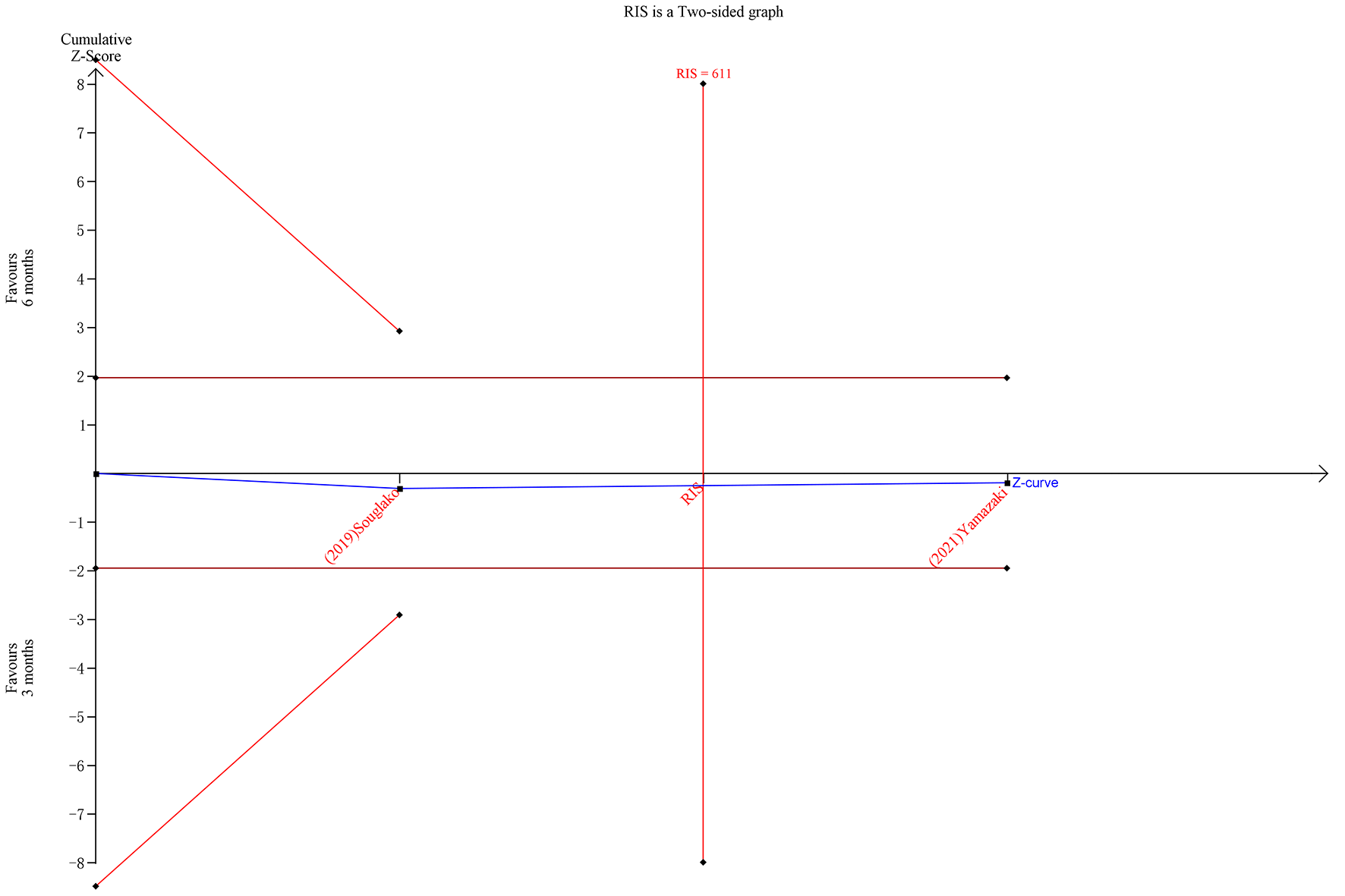

Supplement: Supplementary Figure 9 — TSA results for 3-year DFS rate in stage II patients (CAPOX regimen). Note: The cumulative value exceeds the RIS boundary (RIS = 611). RRR is set to 10%. [file Image9.tif]

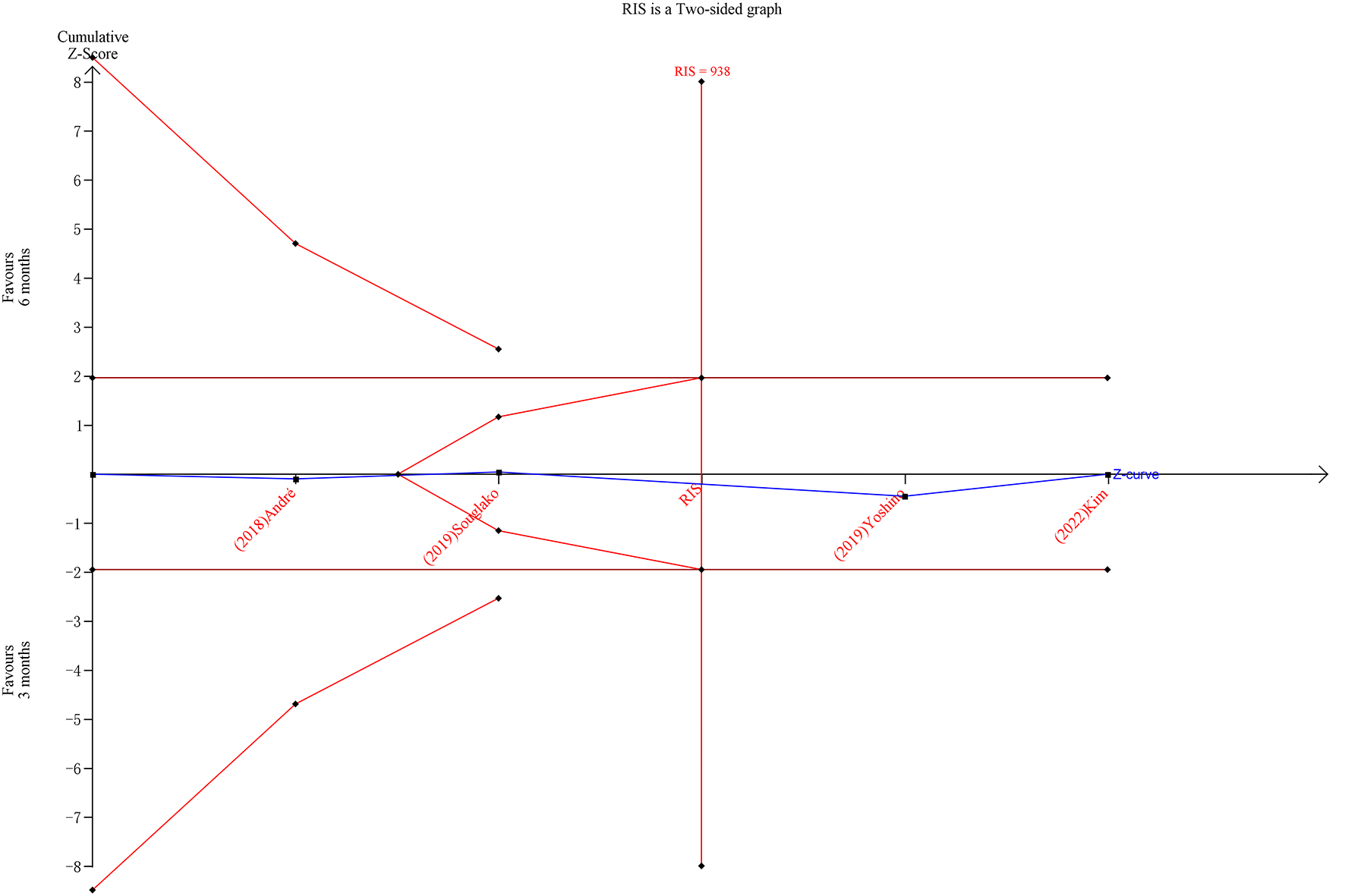

Supplement: Supplementary Figure 10 — TSA results for 3-year DFS rate in stage III patients (CAPOX regimen). Note: The cumulative value exceeds the RIS boundary (RIS = 938). RRR is set to 10%. [file Image10.tif]

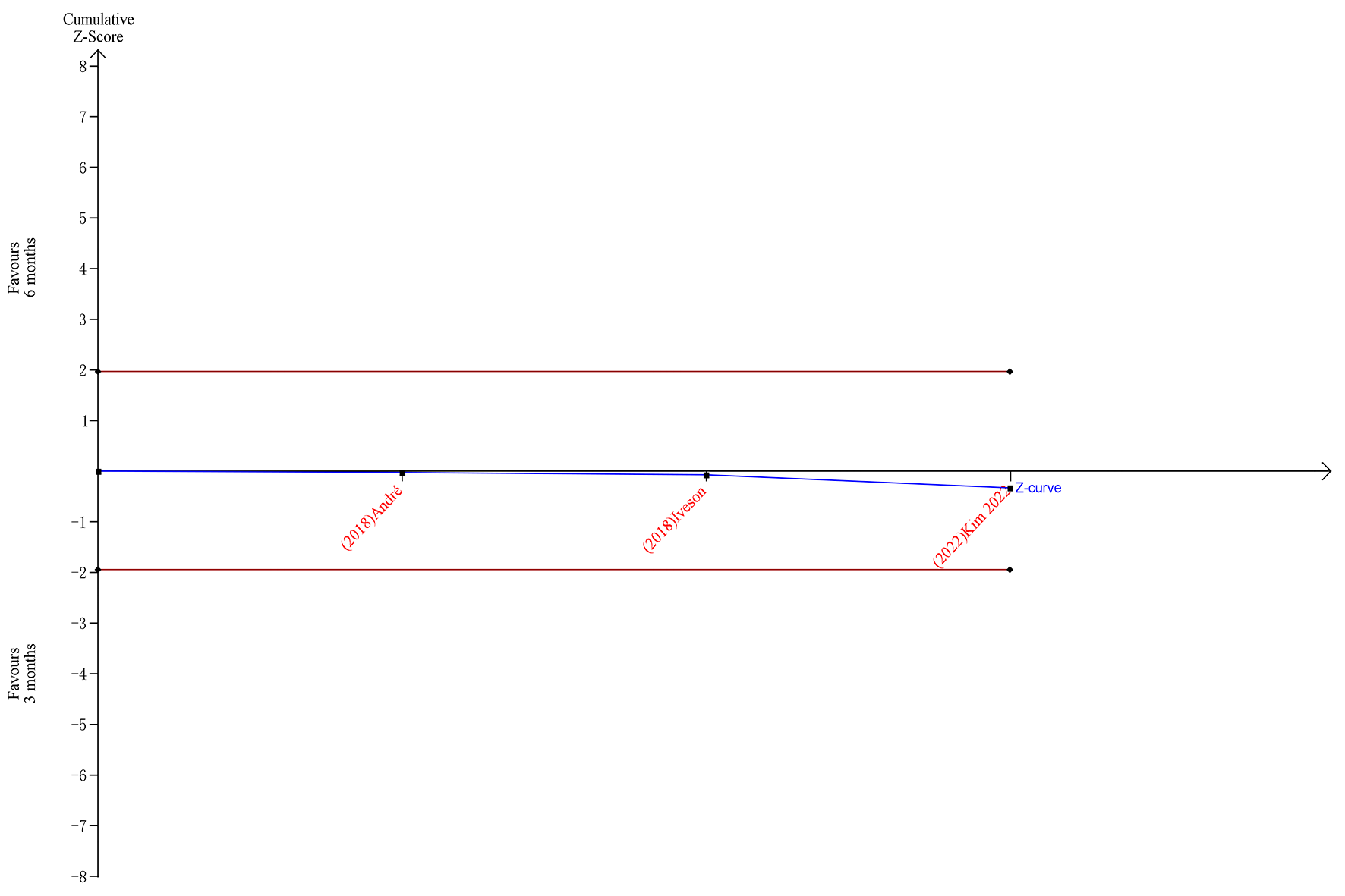

Supplement: Supplementary Figure 11 — TSA results for overall 3-year OS rate (stage II + III). Note: The first study exceeds the RIS boundary (RIS = 496). RRR is set to 10%. [file Image11.tif]

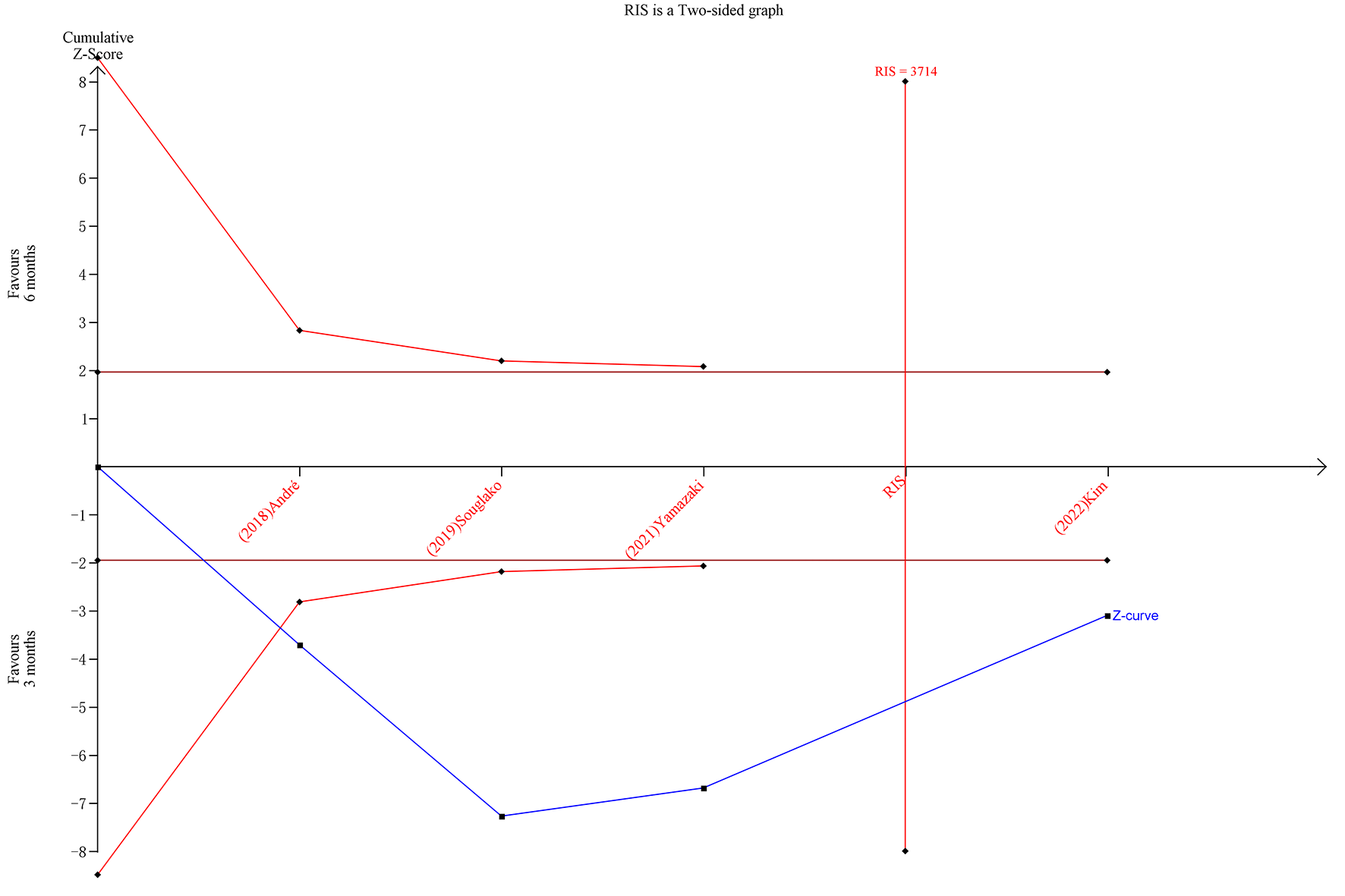

Supplement: Supplementary Figure 12 — TSA results for chemotherapy completion rate. Note: The cumulative value exceeds the RIS boundary (RIS = 3714). RRR is set to 20%. [file Image12.tif]

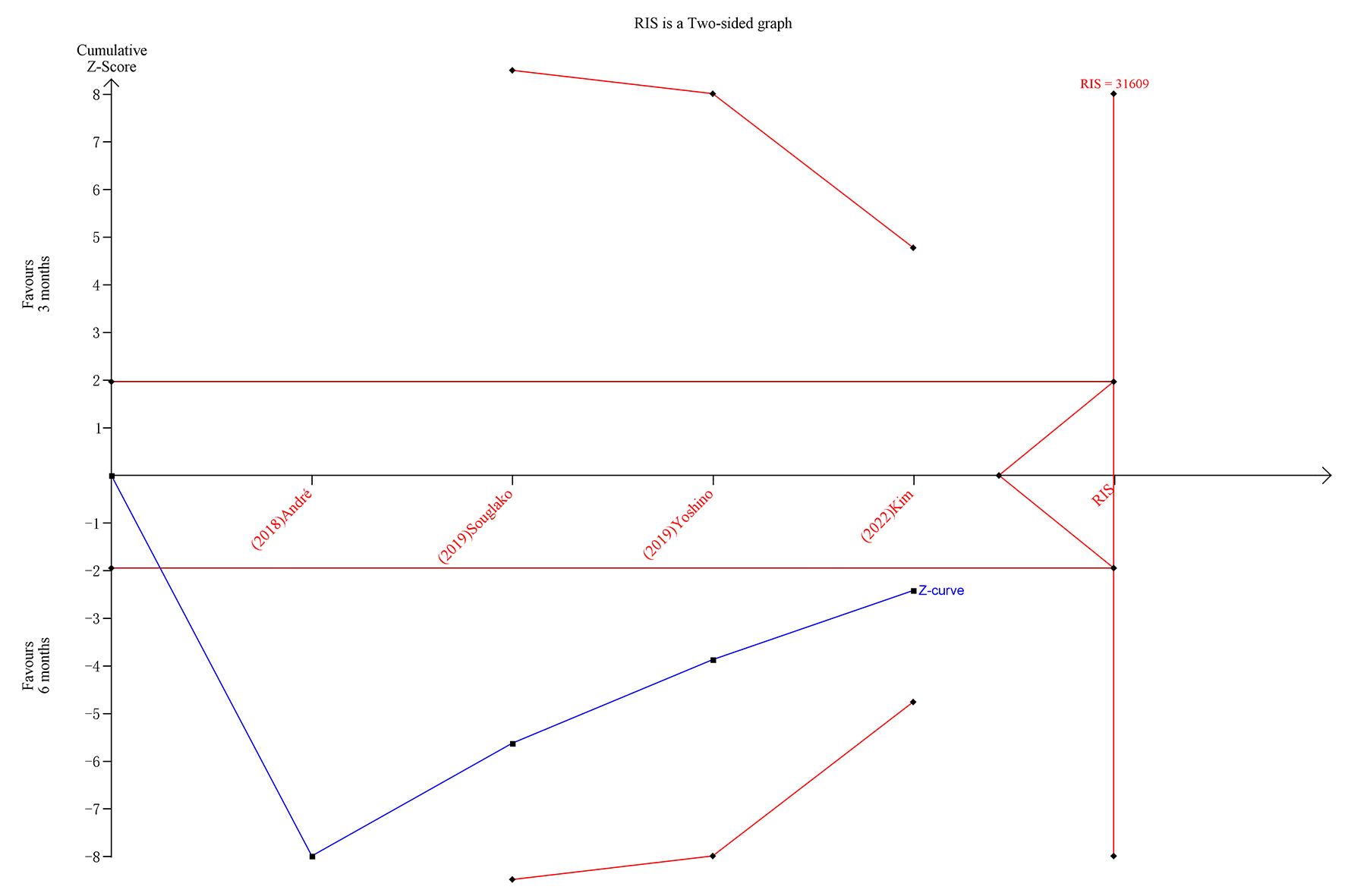

Supplement: Supplementary Figure 13 — TSA results for grade 1 PSN. Note: The cumulative value does not exceed the RIS boundary (RIS = 31609). RRR is set to 20%. [file Image13.tif]

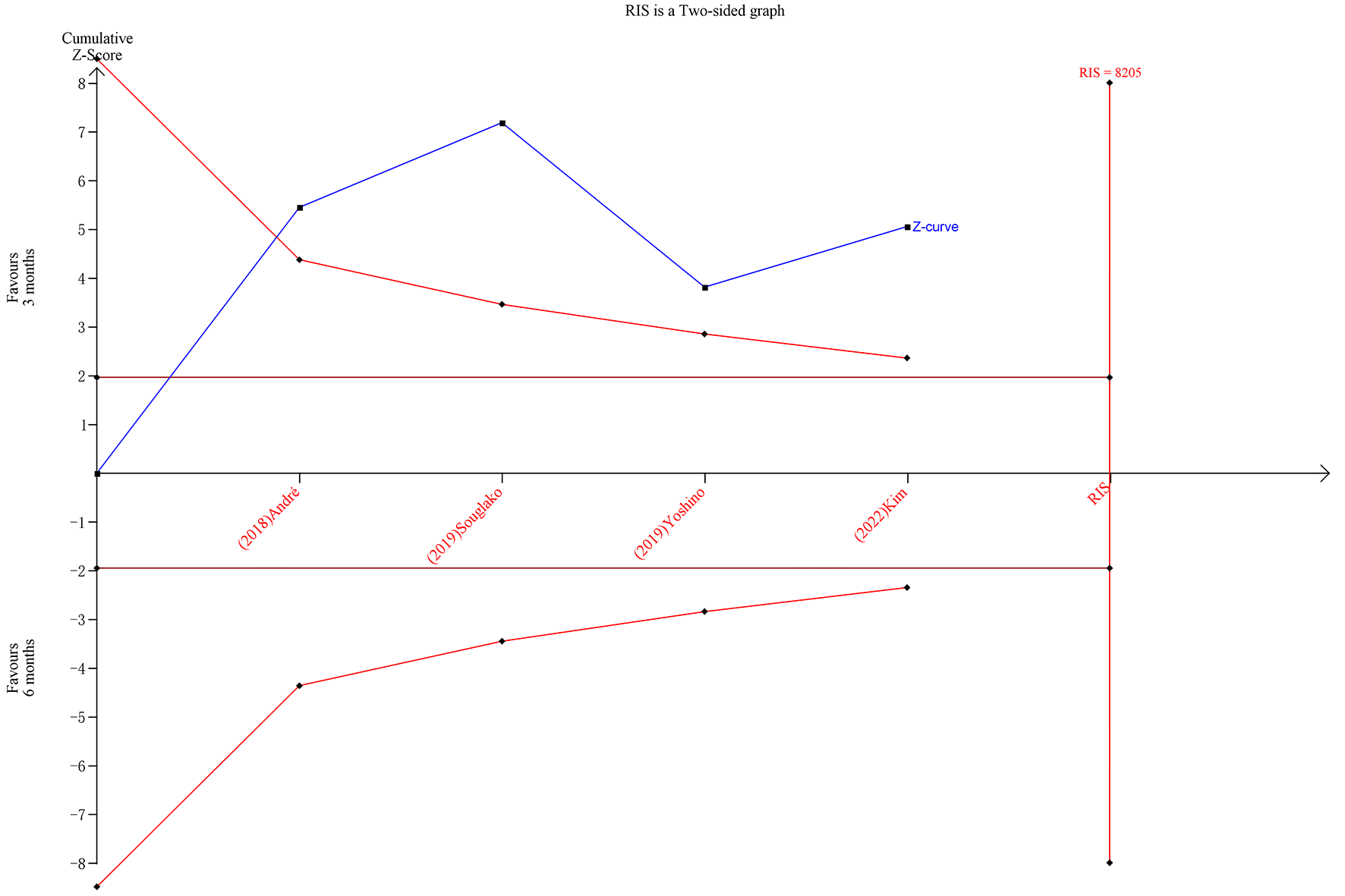

Supplement: Supplementary Figure 14 — TSA results for grade 2 PSN. Note: The cumulative value does not exceed the RIS boundary (RIS = 8205). RRR is set to 20%. [file Image14.tif]

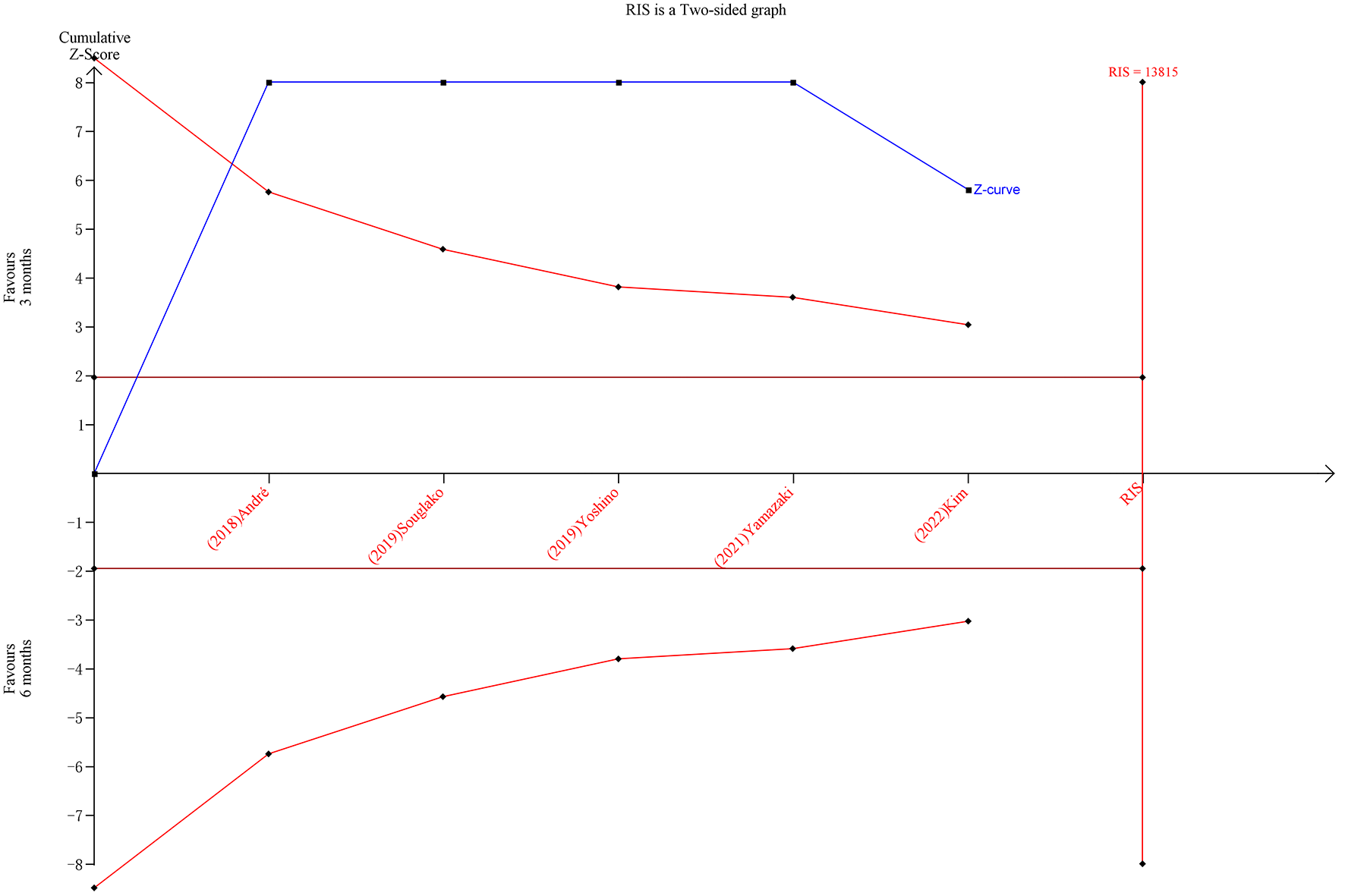

Supplement: Supplementary Figure 15 — TSA results for grade 3–4 PSN. Note: The cumulative value does not exceed the RIS boundary (RIS = 13815). RRR is set to 20%. [file Image15.tif]
