# Supplementary material for: Efficacy and safety of 3-month versus 6-month oxaliplatin-based adjuvant chemotherapy in colorectal cancer: a systematic review and meta-analysis
Source: Front Oncol. 2026 Feb 2;16:1762273. doi: 10.3389/fonc.2026.1762273 (PMC12907182; doi:10.3389/fonc.2026.1762273)
Supplement: Supplementary file 16 [file Supplementaryfile1.docx]

**Supplementary Table 1. GRADE Assessment Results**

| **Quality assessment** | | | | | | | **No of patients** | | **Effect** | | **Quality** | **Importance** |
| --- | --- | --- | --- | --- | --- | --- | --- | --- | --- | --- | --- | --- |
|  |  |  |  |  |  |  |  |  |  |  |  |  |
| **No of studies** | **Design** | **Risk of bias** | **Inconsistency** | **Indirectness** | **Imprecision** | **Other considerations** | **3 months** | **6 months** | **Relative (95% CI)** | **Absolute** |  |  |
| **Overall HR for DFS in the combined stage II + III patients** | | | | | | | | | | | | |
| 6 | randomised trials | serious^1,2,3,4^ | no serious inconsistency | no serious indirectness | no serious imprecision | none^5^ | - | - | HR 1.05 (0.98 to 1.13) | - | ÅÅÅO MODERATE | CRITICAL |
|  |  |  |  |  |  |  |  | 0% |  | - |  |  |
| **Overall 3-year DFS rate in the combined stage II + III patients** | | | | | | | | | | | | |
| 6 | randomised trials | serious^1,2,3,4^ | no serious inconsistency | no serious indirectness | no serious imprecision | none^5^ | 4978/6392  (77.9%) | 5011/6391  (78.4%) | RR 0.99 (0.98 to 1.01) | 8 fewer per 1000 (from 16 fewer to 8 more) | ÅÅÅO MODERATE | CRITICAL |
|  |  |  |  |  |  |  |  | 77.9% |  | 8 fewer per 1000 (from 16 fewer to 8 more) |  |  |
| **HR for DFS in stage II patients** | | | | | | | | | | | | |
| 4 | randomised trials | serious^1,2,3,4^ | no serious inconsistency | no serious indirectness | no serious imprecision | none^5^ | - | - | HR 1 (0.82 to 1.22) | - | ÅÅÅO MODERATE | CRITICAL |
|  |  |  |  |  |  |  |  | 0% |  | - |  |  |
| **3-year DFS rate in stage II patients** | | | | | | | | | | | | |
| 3 | randomised trials | serious^1,2,3,4^ | no serious inconsistency | no serious indirectness | no serious imprecision | none^5^ | 861/1008  (85.4%) | 873/1013  (86.2%) | RR 0.99 (0.96 to 1.03) | 9 fewer per 1000 (from 34 fewer to 26 more) | ÅÅÅO MODERATE | CRITICAL |
|  |  |  |  |  |  |  |  | 87.3% |  | 9 fewer per 1000 (from 35 fewer to 26 more) |  |  |
| **HR for DFS in stage III patients** | | | | | | | | | | | | |
| 4 | randomised trials | serious^1,2,3,4^ | no serious inconsistency | no serious indirectness | no serious imprecision | none^5^ | - | - | HR 1.06 (0.98 to 1.15) | - | ÅÅÅO MODERATE | CRITICAL |
|  |  |  |  |  |  |  |  | 0% |  | - |  |  |
| **3-year DFS rate in stage III patients** | | | | | | | | | | | | |
| 3 | randomised trials | serious^1,2,3,4^ | no serious inconsistency | no serious indirectness | no serious imprecision | none^5^ | 1644/2209  (74.4%) | 1678/2207  (76%) | RR 0.98 (0.95 to 1.01) | 15 fewer per 1000 (from 38 fewer to 8 more) | ÅÅÅO MODERATE | CRITICAL |
|  |  |  |  |  |  |  |  | 76% |  | 15 fewer per 1000 (from 38 fewer to 8 more) |  |  |
| **HR for DFS in high-risk stage II patients** | | | | | | | | | | | | |
| 3 | randomised trials | serious^2,3,4^ | no serious inconsistency | no serious indirectness | no serious imprecision | none^5^ |  |  | HR 1.06 (0.84 to 1.34) |  | ÅÅÅO MODERATE | CRITICAL |
|  |  |  |  |  |  |  |  | 0% |  |  |  |  |
| **3-year DFS rate in high-risk stage II patients** | | | | | | | | | | | | |
| 2 | randomised trials | serious^2,3,4^ | no serious inconsistency | no serious indirectness | no serious imprecision | none^5^ | 245/288  (85.1%) | 257/288  (89.2%) | RR 0.95 (0.9 to 1.01) | 45 fewer per 1000 (from 89 fewer to 9 more) | ÅÅÅO MODERATE | CRITICAL |
|  |  |  |  |  |  |  |  | 87.2% |  | 44 fewer per 1000 (from 87 fewer to 9 more) |  |  |
| **Overall HR for DFS in the combined stage II + III patients (FOLFOX regimen)** | | | | | | | | | | | | |
| 6 | randomised trials | serious^1,2,3,4^ | no serious inconsistency | no serious indirectness | no serious imprecision | none^5^ | - | - | HR 1.21 (1.11 to 1.33) | - | ÅÅÅO MODERATE | CRITICAL |
|  |  |  |  |  |  |  |  | 0% |  | - |  |  |
| **Overall 3-year DFS rate in the combined stage II + III patients (FOLFOX regimen)** | | | | | | | | | | | | |
| 6 | randomised trials | serious^1,2,3,4^ | serious^6^ | no serious indirectness | no serious imprecision | none^5^ | 1912/2551  (75%) | 2074/2575  (80.5%) | RR 0.97 (0.9 to 1.05) | 24 fewer per 1000 (from 81 fewer to 40 more) | ÅÅOO LOW | CRITICAL |
|  |  |  |  |  |  |  |  | 76.5% |  | 23 fewer per 1000 (from 77 fewer to 38 more) |  |  |
| **HR for DFS in stage II patients (FOLFOX regimen)** | | | | | | | | | | | | |
| 2 | randomised trials | serious^1,2,3,4^ | no serious inconsistency | no serious indirectness | serious^7^ | none^5^ | - | - | HR 1.1 (0.55 to 2.18) | - | ÅÅOO LOW | CRITICAL |
|  |  |  |  |  |  |  |  | 0% |  | - |  |  |
| **3-year DFS rate in stage II patients (FOLFOX regimen)** | | | | | | | | | | | | |
| 2 | randomised trials | serious^1,2,3,4^ | no serious inconsistency | no serious indirectness | no serious imprecision | none^5^ | 74/91  (81.3%) | 75/92  (81.5%) | RR 1 (0.87 to 1.15) | 0 fewer per 1000 (from 106 fewer to 122 more) | ÅÅÅO MODERATE | CRITICAL |
|  |  |  |  |  |  |  |  | 81.9% |  | 0 fewer per 1000 (from 106 fewer to 123 more) |  |  |
| **HR for DFS in stage III patients (FOLFOX regimen)** | | | | | | | | | | | | |
| 3 | randomised trials | serious^1,2,3,4^ | no serious inconsistency | no serious indirectness | no serious imprecision | none^5^ | - | - | HR 1.23 (1.06 to 1.43) | - | ÅÅÅO MODERATE | CRITICAL |
|  |  |  |  |  |  |  |  | 0% |  | - |  |  |
| **3-year DFS rate in stage III patients (FOLFOX regimen)** | | | | | | | | | | | | |
| 3 | randomised trials | serious^1,2,3,4^ | no serious inconsistency | no serious indirectness | no serious imprecision | none^5^ | 868/1203  (72.2%) | 922/1218  (75.7%) | RR 0.95 (0.91 to 1) | 38 fewer per 1000 (from 68 fewer to 0 more) | ÅÅÅO MODERATE | CRITICAL |
|  |  |  |  |  |  |  |  | 76% |  | 38 fewer per 1000 (from 68 fewer to 0 more) |  |  |
| **Overall HR for DFS in the combined stage II + III patients (CAPOX regimen)** | | | | | | | | | | | | |
| 6 | randomised trials | serious^1,2,3,4^ | serious^6^ | no serious indirectness | no serious imprecision | none^5^ | - | - | HR 0.98 (0.81 to 1.18) | - | ÅÅOO LOW | CRITICAL |
|  |  |  |  |  |  |  |  | 0% |  | - |  |  |
| **3-year DFS rate in the combined stage II + III (CAPOX regimen)** | | | | | | | | | | | | |
| 6 | randomised trials | serious^1,2,3,4^ | no serious inconsistency | no serious indirectness | no serious imprecision | none^5^ | 2864/3645  (78.6%) | 2829/3620  (78.1%) | RR 1.01 (0.98 to 1.03) | 8 more per 1000 (from 16 fewer to 23 more) | ÅÅÅO MODERATE | CRITICAL |
|  |  |  |  |  |  |  |  | 78.9% |  | 8 more per 1000 (from 16 fewer to 24 more) |  |  |
| **HR for DFS in stage II patients (CAPOX regimen)** | | | | | | | | | | | | |
| 2 | randomised trials | serious^1,2,3,4^ | no serious inconsistency | no serious indirectness | serious^7^ | none^5^ | - | - | HR 1.05 (0.72 to 1.54) | - | ÅÅOO LOW | CRITICAL |
|  |  |  |  |  |  |  |  | 0% |  | - |  |  |
| **3-Year DFS rate in stage II patients (CAPOX regimen)** | | | | | | | | | | | | |
| 2 | randomised trials | serious^1,2,3,4^ | no serious inconsistency | no serious indirectness | no serious imprecision | none^5^ | 323/371  (87.1%) | 323/373  (86.6%) | RR 1.01 (0.95 to 1.06) | 9 more per 1000 (from 43 fewer to 52 more) | ÅÅÅO MODERATE | CRITICAL |
|  |  |  |  |  |  |  |  | 86.2% |  | 9 more per 1000 (from 43 fewer to 52 more) |  |  |
| **HR for DFS in stage III patients (CAPOX regimen)** | | | | | | | | | | | | |
| 4 | randomised trials | serious^1,2,3,4^ | no serious inconsistency | no serious indirectness | no serious imprecision | none^5^ | - | - | HR 0.88 (0.74 to 1.05) | - | ÅÅÅO MODERATE | CRITICAL |
|  |  |  |  |  |  |  |  | 0% |  | - |  |  |
| **3-year DFS rate in stage III patients (CAPOX regimen)** | | | | | | | | | | | | |
| 4 | randomised trials | serious^1,2,3,4^ | no serious inconsistency | no serious indirectness | no serious imprecision | none^5^ | 969/1225  (79.1%) | 953/1204  (79.2%) | RR 1 (0.96 to 1.04) | 0 fewer per 1000 (from 32 fewer to 32 more) | ÅÅÅO MODERATE | CRITICAL |
|  |  |  |  |  |  |  |  | 77.2% |  | 0 fewer per 1000 (from 31 fewer to 31 more) |  |  |
| **Overall HR for OS in the combined stage II + III patients** | | | | | | | | | | | | |
| 4 | randomised trials | serious^1,2,3,4^ | no serious inconsistency | no serious indirectness | no serious imprecision | none^5^ | - | - | HR 1.03 (0.97 to 1.11) | - | ÅÅÅO MODERATE | CRITICAL |
|  |  |  |  |  |  |  |  | 0% |  | - |  |  |
| **Overall 3-year OS rate in the combined stage II + III patients** | | | | | | | | | | | | |
| 3 | randomised trials | serious^1,2,3,4^ | no serious inconsistency | no serious indirectness | no serious imprecision | none^5^ | 4427/4930  (89.8%) | 4420/4933  (89.6%) | RR 1 (0.99 to 1.02) | 0 fewer per 1000 (from 9 fewer to 18 more) | ÅÅÅO MODERATE | CRITICAL |
|  |  |  |  |  |  |  |  | 92% |  | 0 fewer per 1000 (from 9 fewer to 18 more) |  |  |
| **Chemotherapy completion rate** | | | | | | | | | | | | |
| 4 | randomised trials | serious^1,2,3,4^ | serious^6^ | no serious indirectness | no serious imprecision | none^5^ | 2435/2707  (90%) | 2147/2720  (78.9%) | RR 1.16 (1.06 to 1.28) | 126 more per 1000 (from 47 more to 221 more) | ÅÅOO LOW | CRITICAL |
|  |  |  |  |  |  |  |  | 78.7% |  | 126 more per 1000 (from 47 more to 220 more) |  |  |
| **Grade 1 PSN** | | | | | | | | | | | | |
| 4 | randomised trials | serious^1,2,3,4^ | serious^6^ | no serious indirectness | no serious imprecision | none^5^ | 1810/3102  (58.3%) | 1265/3102  (40.8%) | RR 1.41 (1.07 to 1.87) | 167 more per 1000 (from 29 more to 355 more) | ÅÅOO LOW | CRITICAL |
|  |  |  |  |  |  |  |  | 44% |  | 180 more per 1000 (from 31 more to 383 more) |  |  |
| **Grade 2 PSN** | | | | | | | | | | | | |
| 4 | randomised trials | serious^1,2,3,4^ | serious^6^ | no serious indirectness | no serious imprecision | none^5^ | 604/3102  (19.5%) | 999/3102  (32.2%) | RR 0.58 (0.47 to 0.72) | 135 fewer per 1000 (from 90 fewer to 171 fewer) | ÅÅOO LOW | CRITICAL |
|  |  |  |  |  |  |  |  | 32.7% |  | 137 fewer per 1000 (from 92 fewer to 173 fewer) |  |  |
| **Grade 3–4 PSN** | | | | | | | | | | | | |
| 5 | randomised trials | serious^1,2,3,4^ | serious^6^ | no serious indirectness | no serious imprecision | none^5^ | 142/3357  (4.2%) | 457/3361  (13.6%) | RR 0.31 (0.21 to 0.46) | 94 fewer per 1000 (from 73 fewer to 107 fewer) | ÅÅOO LOW | CRITICAL |
|  |  |  |  |  |  |  |  | 7% |  | 48 fewer per 1000 (from 38 fewer to 55 fewer) |  |  |

^1^ The risk of bias for random sequence generation and allocation concealment was unclear.
^2^ Blinding of participants and personnel was not implemented.
^3^ It was not clearly reported whether the outcome assessors were blinded.
^4^ There was insufficient information regarding other potential sources of bias, leading to an unclear risk of bias.
^5^ Not possible to assess due to the small number of included studies (<10).
^6^ Heterogeneity greater than 50%.
^7^ The confidence intervals for the effect estimates are wide.
